# Application of the hybrid ANFIS models for long term wind power density prediction with extrapolation capability

**DOI:** 10.1371/journal.pone.0193772

**Published:** 2018-04-27

**Authors:** Monowar Hossain, Saad Mekhilef, Firdaus Afifi, Laith M. Halabi, Lanre Olatomiwa, Mehdi Seyedmahmoudian, Ben Horan, Alex Stojcevski

**Affiliations:** 1 Power Electronics and Renewable Energy Research Laboratory (PEARL), Department of Electrical Engineering, University of Malaya, Kuala Lumpur, Malaysia; 2 Department of Computer System and Technology, Faculty of Computer Science and Information Technology, University of Malaya, Kuala Lumpur, Malaysia; 3 Department of Electrical and Electronic Engineering, Federal University of Technology, PMB 65, Minna, Nigeria; 4 School of Software and Electrical Engineering, Swinburne University of Technology, Victoria, Australia; 5 School of Engineering, Deakin University, Victoria, Australia; Chongqing University, CHINA

## Abstract

In this paper, the suitability and performance of ANFIS (adaptive neuro-fuzzy inference system), ANFIS-PSO (particle swarm optimization), ANFIS-GA (genetic algorithm) and ANFIS-DE (differential evolution) has been investigated for the prediction of monthly and weekly wind power density (WPD) of four different locations named Mersing, Kuala Terengganu, Pulau Langkawi and Bayan Lepas all in Malaysia. For this aim, standalone ANFIS, ANFIS-PSO, ANFIS-GA and ANFIS-DE prediction algorithm are developed in MATLAB platform. The performance of the proposed hybrid ANFIS models is determined by computing different statistical parameters such as mean absolute bias error (MABE), mean absolute percentage error (MAPE), root mean square error (RMSE) and coefficient of determination (R^2^). The results obtained from ANFIS-PSO and ANFIS-GA enjoy higher performance and accuracy than other models, and they can be suggested for practical application to predict monthly and weekly mean wind power density. Besides, the capability of the proposed hybrid ANFIS models is examined to predict the wind data for the locations where measured wind data are not available, and the results are compared with the measured wind data from nearby stations.

## Introduction

The primary energy sources (fossil fuels) will soon be exhausted since they are used at a much higher rate than they are found in the earth’s crust. Moreover, the price of fossil fuels is highly unstable, and it causes huge greenhouse gases (GHG) emissions and environmental pollutions [1, 2]. On the other hand, the wind energy is free, environmentally friendly and clean renewable energy. Consequently, in the fight of global climate change, wind energy is a major solution [3–5]. Globally, installed wind power capacity has reached 432.9GW at the end of 2015 where 63GW was added in 2015 alone [6].

However, wind energy is unstable and subject to intermittent characteristics thus, the accurate prediction of the wind speed and the wind power is a vital part of the successful establishment of the wind energy conversion system [7]. Again, to build a wind farm in any particular location, analysis of wind data, estimation of wind power and energy density are essential [8, 9]. The wind power density (WPD) of a particular location is the measure of the potentiality of wind resources and the chance of extracting wind energy at different wind speed from that location. The knowledge of WPD also helps the designer and investor to understand the performance of wind turbine and to choose the optimal number of a wind turbine with a suitable power rating [10, 11].

The wind power can be computed from several numerical methods [12, 13]. The problem in numerical methods is that they need high computation time. In the recent years, artificial intelligence (AI) techniques have received overwhelming popularity in the field of the wind energy system and other engineering applications as they offer better advantages, including fast computation time, require no knowledge of internal system parameters and compact solutions [14–20]. Generally, wind speed and power prediction are divided into three categories, namely, short-term (30 min to 6 h), medium-term (6h to 24h), and long-term (24h and longer) predictions [7, 21].

### Short-term wind prediction

In ref. [22], two different short-term wind power prediction methods namely; individual ANN and hybrid strategy based on the physical and statistical methods were developed, where individual ANN and hybrid strategy resulted in 10.67% and 2.01% root mean square error (RMSE) respectively in the prediction. However, the hybrid strategy was 5 times slower than individual ANN. An ANFIS based hybrid model was developed in [23] to predict short-term wind power in Portugal that resulted in MAPE of 5.41%, outperforming five other approaches. The authors used historical wind power data as inputs. In [24], the authors applied both ANN and ANFIS models for hourly wind power prediction for a wind farm located in Southern Italy and their prediction accuracy resulted worse when the prediction horizon was increased. More literature review regarding the application of AI methodologies for the prediction of short-term wind speed and power can be found in the ref. [16, 21].

### Medium-term wind prediction

In [25], an ANN model was employed for the prediction of daily mean wind speed of 11 locations in India where actually measured wind data are not available. The authors used meteorological variables of the target locations from NASA surface meteorology and solar energy database, and the prediction accuracy is compared with measured wind data that was collected from a nearby meteorological station in Hamirpur. A hybrid method consists of wavelet transform (WT), ANFIS, SVM, and GS was proposed in [26] for 6h ahead wind power forecasting. This study showed that the proposed method can predict wind power with MAPE of 12.16% to 13.83%. More literature review regarding the application of soft computing methodologies for the prediction of medium-term wind speed and power can be found in the ref. [21].

### Long-term wind prediction

An ELM model was developed in ref. [27] to predict the monthly wind power density of a particular location in Iran. The authors compared the WPD obtained from ELM model with that from ANN, GP, and SVM. The results showed the performance of ELM higher than other models. The suitability of ANFIS to estimate monthly WPD for the location of Aligoodarz, Iran has been shown in the article [28]. For the prediction of long-term wind speed and power, an ANN and statistical based models were conducted in ref. [7] where the proposed method showed rather promising results in view of the very small mean absolute error (MAE). In study [29], a hybrid model denoted by WT+FA+FF+SVM was reported and the computed MAPE were in the range of 13.46–18.74% for weekly prediction. More literature review regarding the application of soft computing methodologies for the prediction of long-term wind speed and power can be found in the ref. [21, 30].

Long-term prediction of wind speed has become a research hotspot in many different areas such as restructured electricity markets, energy management, and wind farm optimal design. Although ANFIS merges the learning power of the ANNs with the knowledge representation of fuzzy logic, there are still some difficulties in ANFIS in constructing membership functions (MFs). The difficulty of using ANFIS in constructing membership functions lies in tuning the function to build the best model with high accuracy and better performance. Therefore, this study proposed hybrid ANFIS; ANFIS-PSO, ANFIS-GA, and ANFIS-DE to predict long-term (monthly and weekly) wind power density for four different places in Malaysia namely; Mersing, Kuala Terengganu, Pulau Langkawi and Bayan Lepas. The main benefit of combining these three techniques (PSO/GA/DE) with ANFIS is to reduce the error rates by tuning and optimizing the membership functions. Besides, this study examined the wind speed prediction capabilities of the proposed models for the locations where measured wind data are not available, and the result of the wind speed extrapolation is compared with the measured wind data collected from the nearby meteorological station.

## Data collection and analysis

### Site description

The aim of this study is to predict long-term (monthly and weekly) wind power of four different locations situated under four distinctive state of Malaysia shown in Table 1. For this purpose, wind speed data were collected from Malaysian Meteorological Department (MMD) situated in the respective locations during the period (2004–2014). As presented in Table 1, the wind data were measured at different heights above sea level by a rotating cup-type anemometer. Those are 43.6m, 5.2m, 6.4m and 2.46m for Mersing, Kuala Terengganu, Pulau Langkawi and Bayan Lepas respectively. It is important to mention that the wind data recorded in different heights need to adjust to the same height because of various characteristics of wind speed with altitude. The wind shear, which is the variation of wind velocity with altitudes, is most pronounced near the surface (sea and land). Due to the drag of surface and viscosity of air, the wind blows faster at higher altitudes.

**Table 1:** Location and description of the sites.

Locations	State	Geographical coordinate of meteorological stations		Measured height of wind speed (m)
		Latitude	Longitude	
Mersing	Johor	2° 27' N	103° 50' E	43.6
Kuala Terengganu	Terengganu	5° 23' N	103° 60' E	5.2
Pulau Langkawi	Kedah	6° 20' N	99° 44' E	6.4
Bayan Lepas	Pulau Penang	5° 17' N	100° 16' E	2.46

Typically, the variation of wind speed at daytime follows the 1/7^th^ power law whereas, when the temperature become stable or better at night time, the wind speed close to the ground usually subsidies and at turbine altitudes, it does not decrease that much or may even increase. Thus, the daily average wind speed data collected from the meteorological stations were adjusted at turbine hub height of 50m using the power law. The power law for wind speed adjustment at the different hub height is defined as [12]:

$$\frac{v}{v_{o}}{=(\frac{h}{h_{o}})}^{\alpha }$$ (1)

where *v* is the wind speed at is desired height *h* and *v*_*o*_ is the wind speed at measured height *h*_*o*_. While *α* is the power law coefficient. The exponent (*α*) is an empirically derived coefficient that varies depending upon the stability of the atmosphere. For neutral stable conditions, it is approximately 1/7, or 0.143, which is commonly assumed to be constant in wind resource assessments. This is because the differences between the two levels are not usually so great as to introduce substantial errors into the estimation (usually<50m). The value of the coefficient varies from less than 0.10 for very flat land, water or ice to more than 0.25 for heavily forested landscapes and the typical value of 0.14 for low roughness surfaces. The value 0.143 for the coefficient has been chosen for this assessment [12, 31, 32].

### Wind power density (WPD)

The wind power density (WPD) is an essential indicator to estimate wind potentiality in a specific location. The computation methods of WPD include; application of measured wind data and the use of Weibull distribution function. The power in the wind at a measured wind speed *v* passing through a blade sweep area can be expressed as [13, 33]:

$$\bar{P}=\frac{1}{2n}*\rho *\sum_{i=1}^{n}v^{3}=\frac{1}{2}*\rho *\bar{v^{3}}\left(\frac{W}{m^{2}}\right)$$ (2)

where *n* is the number of data points over a time period, $\bar{v^{3}}$ is the mean of cube of wind speed and *ρ* is the air density (*kg*/*m*^3^), taken 1.175 *kg*/*m*^3^ in this study.

As presented in [12, 31], the 2-parameters Weibull distribution function is the most appropriate, recommended and acceptable model for wind potentiality analysis. In Weibull distribution, the probability distribution function (PDF) determines the probability of the wind at a given velocity *V* and it can be expressed as [12, 34]:

$$f(V)=\left(\frac{k}{c}\right){\left(\frac{V}{c}\right)}^{k-1}exp\left[{-(\frac{V}{c})}^{k}\right]$$ (3)

On the other hand, cumulative distribution function (CDF) of wind speed *V* indicates the probability that the wind velocity is equal to or lower than *V* or within a given wind speed range. CDF can be expressed as [12, 34]:

$$F(V)=1-exp\left[{-(\frac{V}{c})}^{k}\right]$$ (4)

where *V* is wind speed (m/s), k (dimensionless) is shape factor and c (m/s) is scale factor.

As earlier mentioned, the k and c (m/s) can be computed by several empirical methods. Commonly used is standard deviation method, k and c are defined as follows [12, 13]:

$${k=(\frac{\sigma }{\bar{v}})}^{-1.086}$$ (5)

$$c=\frac{\bar{v}}{\Gamma (1+\frac{1}{k})}$$ (6)

where $\bar{v}$ is the average wind speed (m/s), *σ* is standard deviation and *Γ*(*x*) is the gamma function which is defined as [35, 36]:

$$\Gamma (x)=\int_{0}^{\infty }t^{x-1}e^{-t}dt$$ (7)

Another empirical method is power density or energy pattern factor method. In this method, *E*_*pf*_ is needed to be estimated to compute the shape factor k and scale factor c (m/s).

The *E*_*pf*_ is known as wind pattern factor which is used for wind turbine aerodynamic design and it is defined as follows [33, 37]:

$$E_{pf}=\frac{\frac{1}{n}\sum_{i=1}^{n}{v_{i}}^{3}}{{\left(\frac{1}{n}\sum_{i=1}^{n}v_{i}\right)}^{3}}=\frac{\bar{v^{3}}}{{\bar{v}}^{3}}=\frac{\Gamma (1+\frac{3}{k})}{\Gamma^{3}(1+\frac{1}{k})}$$ (8)

In simple word, *E*_*pf*_ is the ratio of mean of cube wind speed to cube of mean wind speed. When *E*_*pf*_ is known then shape factor k can be easily estimated by following formula [33, 37]:

$$k=1+\frac{3.69}{{\left(E_{pf}\right)}^{2}}$$ (9)

The wind power density on the basis of Weibull probability density function is estimated using the following equation [13]:

$$\bar{P}=\frac{1}{2}\;\rho \;\int_{0}^{\infty }v^{3}f_{w}(v)dv=\frac{1}{2}{\rho \;c}^{3}\Gamma \left(1+\frac{3}{k}\right)\;(W/m^{2})$$ (10)

## Methodology

As mentioned in section 2.1, to accomplish the study objective, 11 years (2004–2014) long-term wind speed data measured by the rotating cup-type anemometer were collected from the four different locations in Malaysia; Mersing, Kuala Terengganu, Pulau Langkawi and Bayan Lepas. The raw data was thereafter adjusted to turbine hub height of 50m. The raw data used in this study is presented in S1 Dataset: Supporting Information.rar. Afterward, the monthly and weekly mean wind speed at 50m and the corresponding wind power density from measured data were applied on the developed standalone ANFIS and hybrid ANFIS models. In this study, the data size used for the training and testing the prediction models are defined as P and Q respectively. The purpose of the training process in ANFIS model is to minimize the error between the actual target and the ANFIS output. Based on the literature on ANN, the percentage of training data must be higher than testing data for effective learning of the system before the system can produce a good result. The developed models were trained and validated with several data segments such as P = 80%, Q = 20%; P = 70%, Q = 30% and P = 60%, Q = 40% for training and testing respectively. The percentage of data selected for training and testing has been carefully tested based on the minimal error obtained in the statistical indicator. It is important to mention that no specific rules were considered to choose the data size for training and testing the models. Application of different training and testing data size on the prediction models helps to observe the error metrics and to choose best data size providing the minimum error in the prediction.

### ANFIS (adaptive neuro-fuzzy inference system)

**Fig 1:**
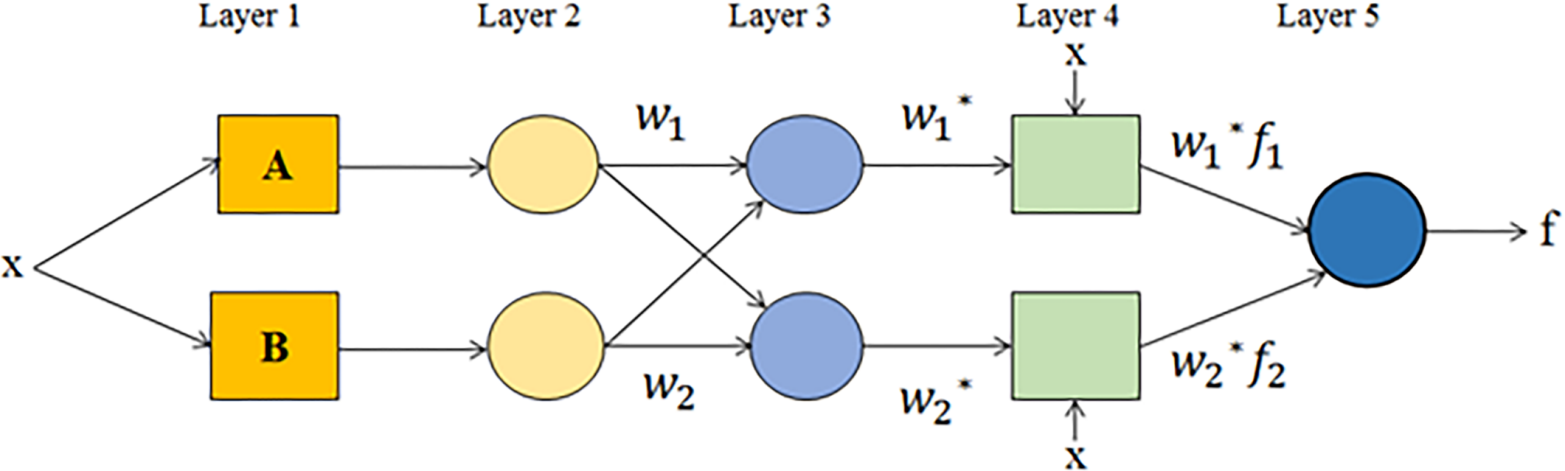
ANFIS structure.

The term adaptive neuro-fuzzy inference system (ANFIS) was first introduced by Jang in 1993. ANFIS is a hybrid intelligent scheme that merges the learning power of ANNs with the knowledge representation of fuzzy logic to produce a powerful processing tool [38]. The fuzzy inference system (FIS) is the core of ANFIS. FIS is based on expertise expressed in terms of ‘IF–THEN’ rules, thus it can be used to predict the behavior of many uncertain systems. One of the advantages of FIS is that it does not require knowledge of the main physical process as a pre-condition for operation. Thus, ANFIS integrates FIS with a back propagation learning algorithm of a neural network. These techniques provide a method for the fuzzy modeling procedure to learn from the available data set, in order to compute the membership function parameters that best allow the associated fuzzy inference system to track the given input/output data as shown in Fig 1. For one input, two fuzzy ‘IF-THEN rule are generated for the maximum equal to 1 and minimum equal to 0. The fuzzy inference system employed in this study uses one input *x* and one output *f*. A first-order Sugeno fuzzy model with two fuzzy *if-then* rules is used as follows [39]:

$$Rule\;1:\;if\;x\;is\;A\;then\;f_{1}=p_{1}x+t_{1}$$ (11)

$$Rule\;2:\;if\;x\;is\;B\;then\;f_{2}=p_{2}x+t_{2}$$ (12)

Layer 1 contains membership functions (MFs) of input variables and provides the input values for the next layer. In the 1st layer, each node is adaptive as *O* = *μ*_*AB*_(*x*), where *μ*_*AB*_(*x*) are MFs. The bell-shaped MFs is presented in Eq 13, for which the lowest and highest amounts are 0 and 1, respectively.

$$f(x;a,b,c)=\frac{1}{1+{\left(\frac{x-c}{a}\right)}^{2b}}$$ (13)

**Fig 2:**
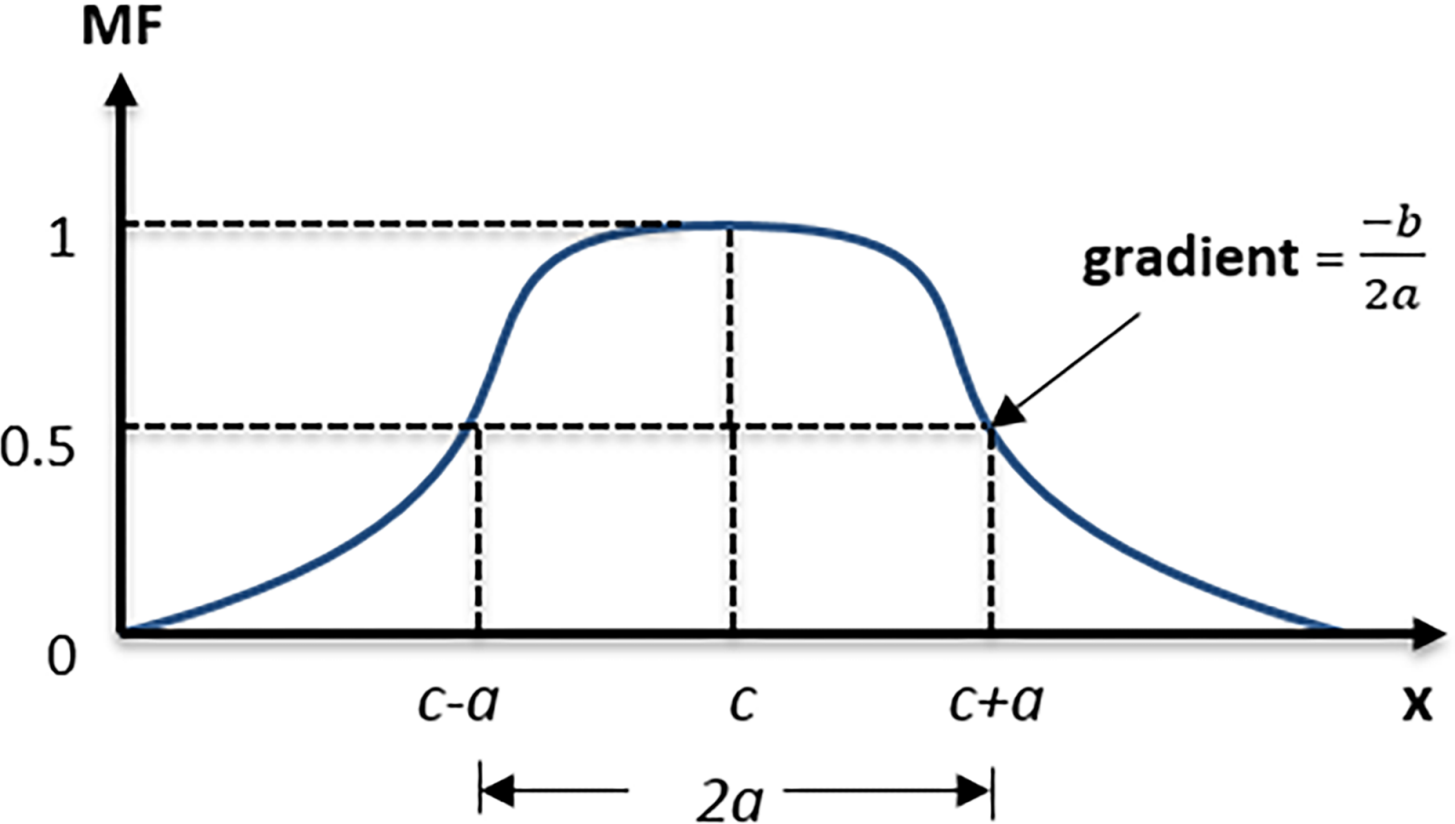
Three parameters bell-shaped membership function; *a*, *b* and *c*.

The function is subject to the following parameters, namely *a*, *b* and *c*. Each of these parameters defines as follows: *a* is half width of the curve; *b* define the gradient together with *a*, and *c* is the midpoint of the membership function as shown in Fig 2.

In the 2nd layer (the membership layer), the weight of membership functions (MFs) is considered. Input values for this layer are supplied from the first layer. The nodes in the second layer are a fixed node. The output is the product of all incoming signals and be described as,

$$w_{i}={\mu (x)}_{i}∙{\mu (x)}_{i+1},\;for\;i=1,2$$ (14)

The output of each node indicates the weight strength of a rule.

In layer 3 or the rule layer, every node does the pre-condition matching of the fuzzy rules, that calculate each rule’s activation level as well as the normalized firing strength. This is a fixed layer as well, and every node computes the ratio of the *i*th rule of the firing strength to the sum of *i*th firing strengths of all rules as:

$$w_{i}^{*}=\frac{w_{i}}{w_{1}+w_{2}},\;for\;i=1,2$$ (15)

The outputs of this layer are named as normalized weights or firing strengths.

In layer 4 or defuzzification layer, all the adaptive nodes provide the resulting output values from the inference of rules.

$$O_{i}^{4}=w_{i}^{*}∙f=w_{i}^{*}(p_{i}x+t_{i})$$ (16)

Here, the parameters set are shown as {*p*_*i*_,*t*}.

Layer 5 or the output layer summarizes the inputs and output from layer 4. This layer also converts the results of fuzzy classification into a crisp. Here, the single node is fixed node, and the whole incoming signal is sum up to produce overall output as below,

$$O_{i}^{5}=\sum_{i}w_{i}^{*}∙f=\frac{\sum_{i}w_{i}∙f}{\sum_{i}w_{i}}$$ (17)

Three optimization techniques namely; PSO, GA, and DE were employed to adjust the ANFIS membership function parameters. The main benefit of combining these three techniques with ANFIS is to reduce the error rates by tuning and optimizing the membership functions.

### ANFIS-PSO

Particle swarm optimization (PSO) is an approach for optimizing “continuous” and “discontinuous” decision-making functions as developed by Kennedy and Eberhart in 1995 [40]. PSO has been used to model animals’ sociological and biological behavior such as groups of birds searching for food [41]. PSO has also been employed in population-based search approach, in which a particle of a population is present for each individual potential solution or swarm. In this method, the position of each particle is changed constantly in a search space until getting to the optimum solutions and computational limitations are reached [42]. In PSO, swarm starts with a group of random solutions, each of which is called a particle, and ${\overset{⃑}{s}}_{i}$ represents the particle’s position. Likewise, a particle swarm moves in the problem space, where ${\overset{⃑}{v}}_{i}$ expresses the particle’s velocity. A function *f* is evaluated at each time step through input ${\overset{⃑}{s}}_{i}$. Every particle records its best position related to the best fitness gained to this point, in ${\overset{⃑}{p}}_{i}$ vector. ${{\overset{⃑}{p}}_{i}}^{g}$ tracks the most appropriate position identified by any neighborhood member. In universal form of PSO, ${{\overset{⃑}{p}}_{i}}^{g}$ represents the best appropriate position in the entire population. A new velocity is achieved for any particle *i* in each iteration according to the best positions of individual, ${\overset{⃑}{p}}_{i}(t)$ and ${\overset{⃑}{p}}_{i}^{g}(t)$ neighborhood. The new velocity can be presented by:

$${\overset{⃑}{v}}_{i}(t+1)=w{\overset{⃑}{v}}_{i}(t)+c_{1}\overset{⃑}{\emptyset_{1}}.({\overset{⃑}{p}}_{i}(t)-{\overset{⃑}{x}}_{i}(t))+c_{2}\overset{⃑}{\emptyset_{2}.}({\overset{⃑}{p}}_{i}^{g}(t)-{\overset{⃑}{x}}_{i}(t))$$ (18)

where *w* represents the inertia weight and positive acceleration coefficients are represented by *c*_1_ and *c*_2_. $\overset{⃑}{\emptyset_{1}}$ and $\overset{⃑}{\emptyset_{2}}$ represent uniformly-distributed random vectors [0,1], in which a random value is tried for every dimension. ${\overset{⃑}{v}}_{i}$ limit in the [$\text{-}\overset{⃑}{v_{max}},\;\overset{⃑}{v_{max}}$] range is relent on the problem provided the velocity exceeds the mentioned limit. In some cases, it is rearranged within its suitable limits. The position of every particle alters depending upon the velocities as:

$${\overset{⃑}{s}}_{i}(t+1)={\overset{⃑}{s}}_{i}(t)+{\overset{⃑}{v}}_{i}(t+1)$$ (19)

**Fig 3:**
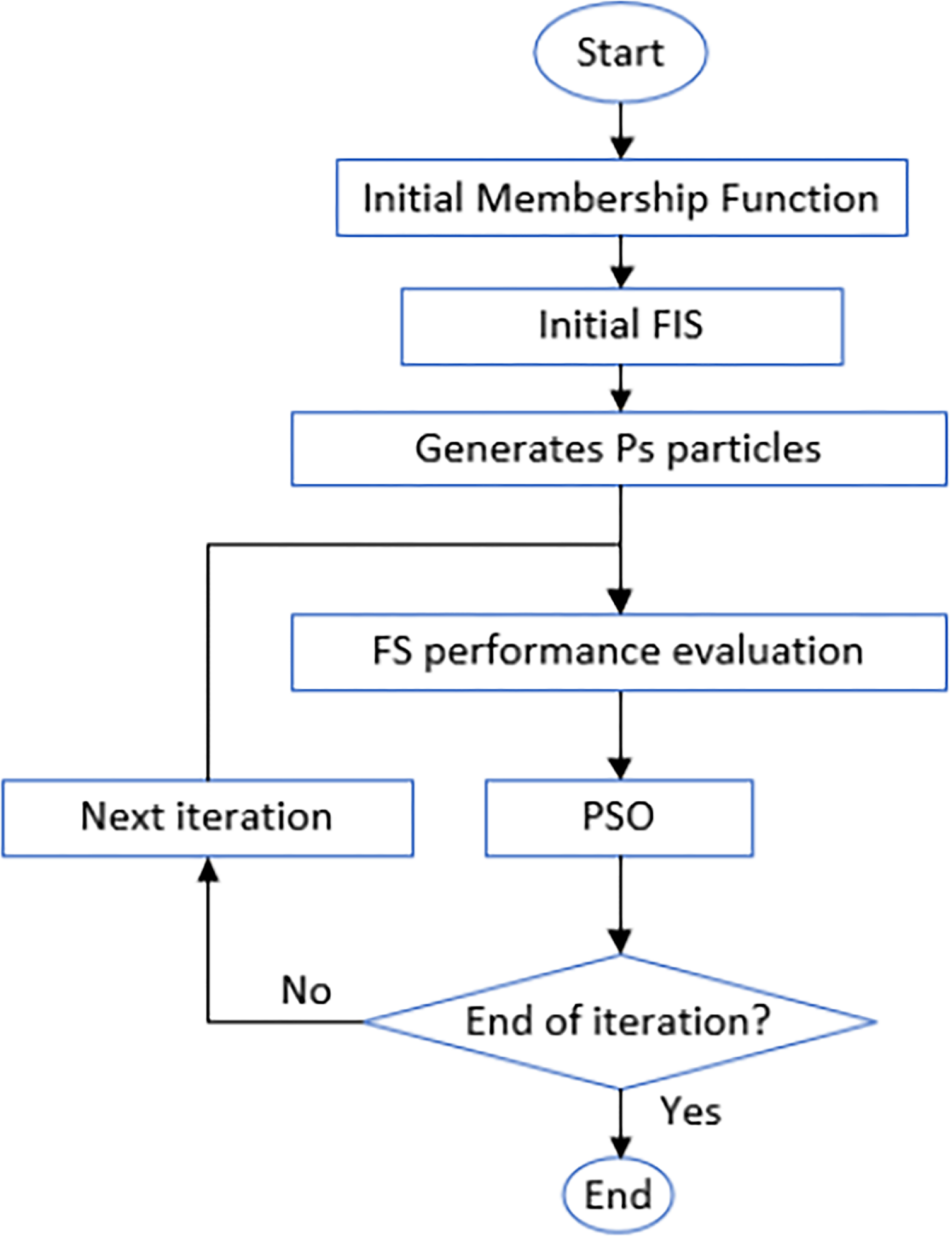
The sequential combination of PSO and ANFIS.

According to Eqs (18) and (19), the particles incline to gather around the best. Fig 3 depicts the sequential PSO and ANFIS combination [43].

The PSO use for designing a fuzzy system (FS) or parameter optimization is expressed as [44]:

$$R_{i}:\;if\;x_{1}(k)\;is\;A_{i1}\;And\;\ldots \;And\;x_{n}(k)\;is\;A_{in}\;,\;Then\;u(k)\;is\;a_{i}$$ (20)

where, *a*_*i*_ is a crisp value, *k* represents the time step, the input variables are *x*_1_(*k*),…,*x*_*n*_(*k*), *A*_*ij*_ is a fuzzy set *and u*(*k*) signifies the system output variable.

For the FS in Eq (20) which comprises *r* rules and *n* input variables, it’s free parameters are defined through a position vector:

$$\overset{⃑}{s}=[m_{11},b_{11},\ldots ,m_{1n},b_{1n},a_{1},\ldots \ldots ,m_{r1},b_{r1},\ldots ,m_{rn},b_{rn},a_{r}]\in R^{D}$$ (21)

$$m_{rj}=x_{j}(k),b_{rj}=b_{fix},\;j=1,\ldots ,n$$ (22)

Following the process of rule creation and initialization, the preliminary antecedent part parameters are outlined. According to Eq (21) and Eq (22), the *i*th solution vector $\overset{⃑}{s_{i}}$ is created as:

$$\overset{⃑}{s_{i}}=[s_{i1}\;s_{i2}\ldots s_{iD}]=[m_{11}+\Delta m_{11}^{i},b_{fix}+\Delta b_{11}^{i},\ldots ,m_{1n}+\Delta m_{1n}^{i},b_{fix}+\Delta b_{1n}^{i},a_{1},\ldots ,m_{r1}+\Delta m_{r1}^{i},b_{fix}+\Delta b_{r1}^{i},\ldots ,m_{rn}+\Delta m_{rn}^{i},b_{fix}+\Delta b_{rn}^{i},a_{r}]$$ (23)

In Eq (23), Δ*m*_*ij*_ and Δ*b*_*ij*_ represent small random numbers, *a*_*i*_ designates a random number distributed arbitrarily and homogeneously in the FS output range. The *f* (evaluation function) for $\overset{⃑}{s_{i}}$ is calculated based upon the FS performance.

PSO looks for the best originator part parameters. *P*_*S*_ represents the population size. Eq (21) sets the elements in position $\overset{⃑}{s}$. When *t* = 0, the $\overset{⃑}{s_{1}}(0),\ldots ,\overset{⃑}{s_{p}}(0)$ or initial positions are created arbitrarily according to the best-performing FS found in PSO (${\overset{⃑}{s}}_{PSO}$). $\overset{⃑}{s_{1}}(0)$ is considered similar to ${\overset{⃑}{s}}_{PSO}$. The left *P*_*S*_ − 1 particles, $\overset{⃑}{s_{2}}(0),\ldots ,\overset{⃑}{s_{p_{s}}}(0)$, are created by addition of uniformly-distributed random numbers to ${\overset{⃑}{s}}_{PSO}$ shown as:

$$\overset{⃑}{s_{i}}(0)={\overset{⃑}{s}}_{PSO}+\overset{⃑}{w_{i}},\;i=2,\ldots ,P_{s}$$ (24)

where, $\overset{⃑}{w_{i}}$ represents a random vector. The primary speed values of all particles, $\overset{⃑}{v_{i}}(0),\;i=1,\ldots ,P_{s}$, are generated randomly. Each particle’s performance is evaluated according to the FS it signifies. *f* is described as the *E*(*t*) or error index mentioned above. The best position ($\overset{⃑}{p_{i}}$) of each particle and the best particle $\overset{⃑}{p_{g}^{i}}$ in the whole population is obtained according to *f*. Eqs (18) and (19) overhaul the velocity and position of each particle. The whole learning procedure is accomplished as soon as a pre-defined paradigm is obtained [44].

There are five PSO main parameters used during conducting the experiment as shown in Table 2, this includes; a maximum number of iterations, the population size of the domain, inertia weight damping ratio and inertia weight, global and personal learning coefficient. For this case studies, the optimum values of these parameters are determined by trial and error procedure.

**Table 2:** Parameter characteristics for ANFIS-PSO.

Population Size	Iterations	Inertia Weight	Damping Ratio	Learning coefficient	
				Personal	Global
100	500	1	0.99	2	2

### ANFIS-GA

Genetic Algorithm is global search heuristics technique used to find solutions for optimization and solve highly complex search problems. It is a particular class of evolutionary computing method inspired by the idea of natural selection evolutionary process which implemented inheritance, mutation, selection, and recombination [45]. In this hybrid approach, GA is combined with ANFIS to extend its prediction proficiency. GA is implemented to improve ANFIS performance and minimize the error rates by tuning and optimizing the membership functions of a Sugeno type fuzzy inference system. The ANFIS-GA forecast allows reforming of the upcoming behavior of the wind power density and therefore, determines the viability of the wind power plants from any location.

**Fig 4:**
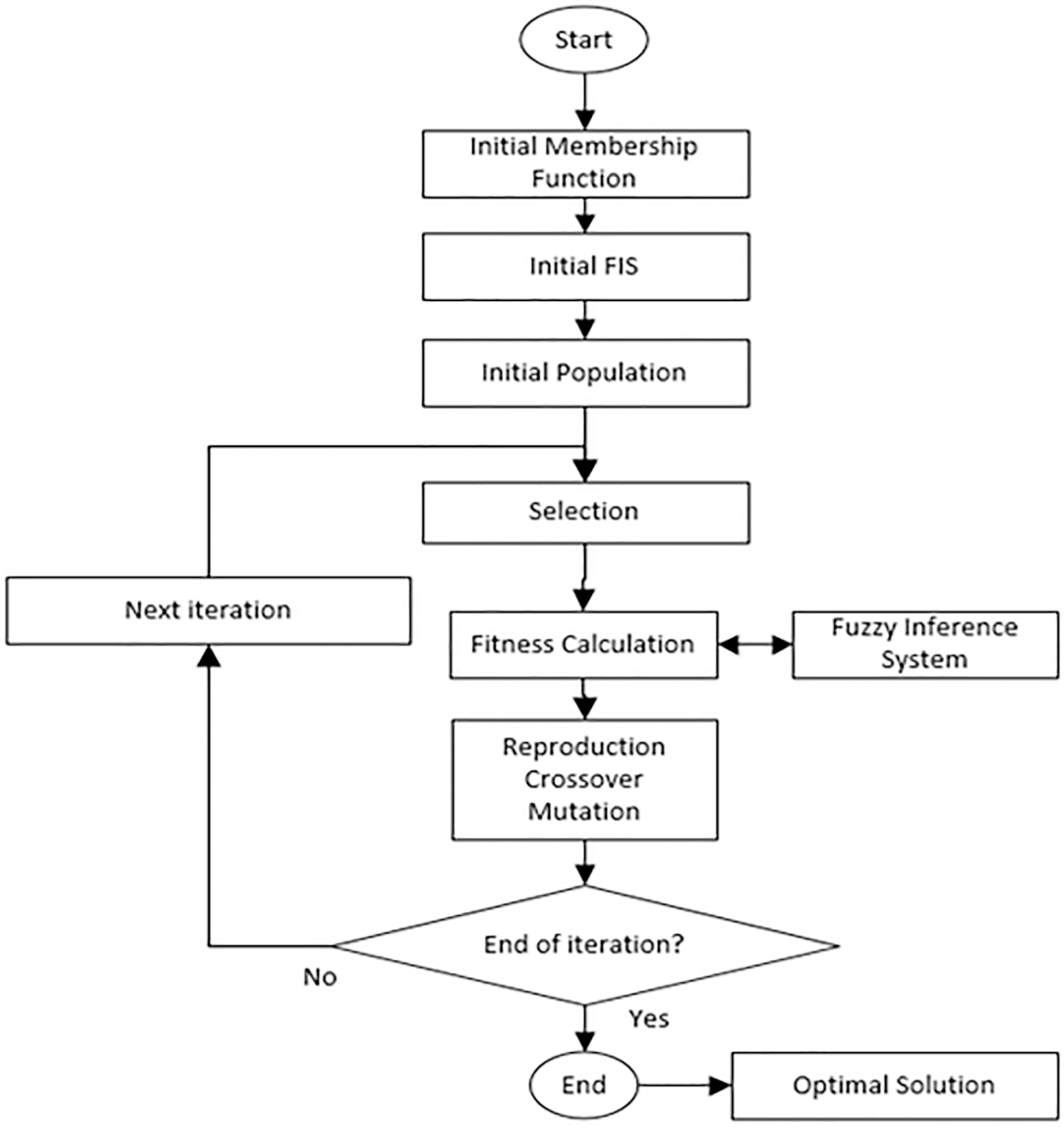
ANFIS-GA model.

The Hybrid ANFIS-GA model used is shown in Fig 4 [46].

GA model begins with a set of solutions (referred to as chromosomes) represented as population. A new population is drawn from the completion of a previous population. New solutions that formed from selected solution (offspring) are designated according to their fitness.

This process is repeated until some condition is occurred (for example, number of populations or improvement of the optimal solution) is fulfilled. To achieve this, ANFIS algorithm plays an important role as part of the fitness function, *f*(*x*). The fitness with intervention of ANFIS fitness function is represented by;

$$f_{1}(x)=\frac{1}{m}\sqrt{\sum_{i=0}^{m}{\left(d_{i}-a_{i}\right)}^{2}}$$ (25)

where *m* is a number of feature attributes, *a*_*i*_ is output derived through ANFIS, *d*_*i*_ is desired wind power density.

The next fitness function can be presented as:

$$f_{2}(x)=\frac{1}{n-m}\sqrt{\sum_{i=m}^{n}{\left(d_{i}-a_{i}\right)}^{2}}$$ (26)

where *n* is the total number of input features, *d*_*i*_ is set to minimum, *a*_*i*_ is actual value of wind power density and *n* − *m* represents remaining undesired features. The final equation is minimized *f*(*x*), describe as,

$$f(x)=\frac{f_{1}(x)+f_{2}(x)}{2}$$ (27)

For this case study, we determined the specific parameters initialization for the GA. These include; number of iterations, population size, mutation and crossover percentage. Selection of these parameters decides, to a great extent, the ability of the designed controller. The range of the tuning parameters is listed in Table 3.

**Table 3:** Parameter characteristics of ANFIS-GA.

Population Size	Number of Iterations	Crossover Percentage	Mutation Percentage	Mutation Rate	Selection Pressure	Selection Function
100	500	0.8%	0.3%	0.02	8	Roulette Wheel

After the fitness *f*(*x*) of each chromosome *x* in the population is evaluated, new population is created and following steps are repeated until it is completed. The better fitness gives bigger chance to parent chromosomes from a population to be selected. Then it is crossover the parents to form a new offspring with the crossover probability. Next mutation probability mutates new offspring at each position in chromosome. As the solution goes under Reproduction, Crossover and Mutation with parameters setting from 3, the best solution in current population is returned if end condition is satisfied. The optimal solution calculated will help GA to search for optimized membership function.

### ANFIS-DE

Differential evolution (DE) is first introduced as a heuristic method by Storn and Price [47] to solve problems involving global optimization as the solution to minimizing possibly nonlinear and non-differentiable continuous space functions. Since both DE and GA are part of evolutionary computing methodologies, DE functions almost in the same manner as GA. The different in DE is using actual real numbers in a strict mathematical sense, which can be applied to real-valued problems over a continuous space. As a result, the designs of crossover and mutation are significantly different. The idea behind the method of DE is that the difference between two vectors produces a difference vector which is used with a scaling factor to traverse the search space [48].

**Fig 5:**
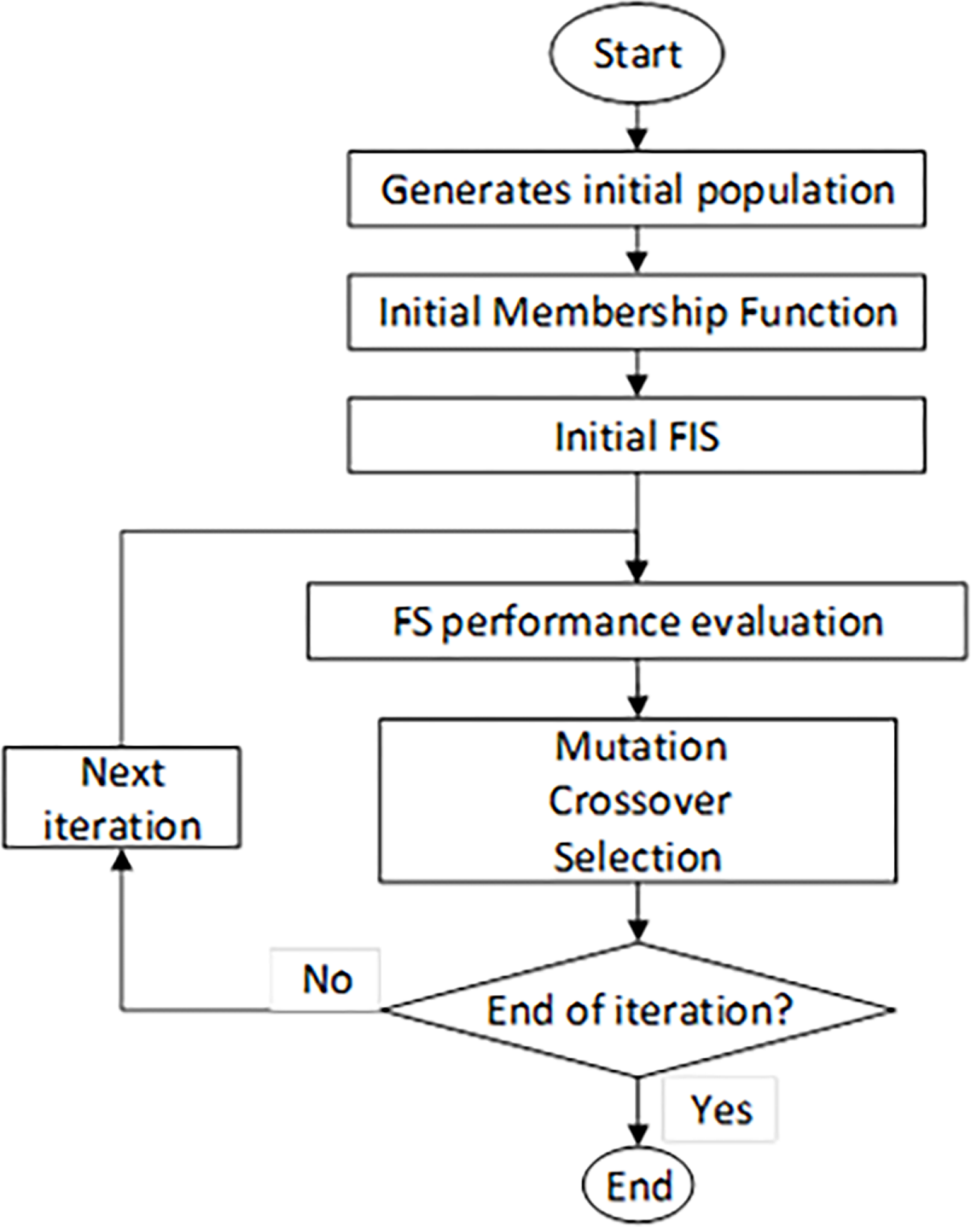
ANFIS-DE model.

In ANFIS-DE hybrid approach, DE is combined with ANFIS to improve the performance of ANFIS prediction proficiency. Differential Evolution initialize with population size of (*pop size*) individuals solutions which can be represented as $x_{i}^{t}=1,2,\ldots ,popsize$ for each individual where *i* represents the population and *t*_*th*_ represents the generation to which the population belongs. Then the algorithm depends on the operation of three main operators; mutation, crossover and selection as shown in Fig 5 [46].

Mutation operator is the main operator of DE which differs from other EAs. We implemented DE/rand/1 mutation strategy as described in Eq 28.

$$u_{i}^{t}=x_{r_{1}}^{t}+F.(x_{r_{2}}^{t}-x_{r_{3}}^{t})$$ (28)

where $u_{i}^{t}$ is the mutant individual for $x_{i}^{t}$ and *r*_1_,*r*_2_,*r*_3_ are randomly selected and satisfy individuals. Moreover, they are not equal to running index (*i*) and mutually different. *F* is control parameter in the range of [0, 2]

Process crossover is carried out after the mutation phase is completed and can be described as,

$$y_{id}^{t}=\left\{ \begin{array}{l} u_{id}^{t}\;if\;rand\leq C_{R},\\ x_{id}^{t}\;otherwise, \end{array}\right.$$ (29)

where $y_{i}^{t}$ the trial individual, d is representing the *d*_*th*_ component of individuals. The *rand* and crossover rate *C*_*R*_ is a parameter in the range of [0, 1].

Selection operation in DE is also different from other evolutionary algorithms. Once all *pop size* trial individuals are generated, the selection operation is processed as:

$$x_{id}^{t+1}=\left\{ \begin{array}{l} y_{i}^{t}\;if\;fitness\;(y_{i}^{t})\leq fitness\;(x_{i}^{t}),\\ x_{i}^{t}\;otherwise, \end{array}\right.$$ (30)

where *fitness*(*x*) is the fitness function. Table 4 shows the initial parameters of the ANFIS-DE model.

**Table 4:** Parameter characteristics for ANFIS-DE.

Population Size	No. of Iterations	Crossover Percentage	Lower bound of scaling factor	Upper bound of scaling factor
100	500	0.2%	0.2	0.8

## Statistical indicators model performance evaluation

The performance of the proposed system can be checked by computing several statistical parameters. The most popular statistical error indicators are the mean absolute bias error (MABE), mean absolute percentage error (MAPE), root mean square error (RMSE) and coefficient of determination (R^2^). The MABE is the average quantity of the summation of all absolute bias error between the predicted and the measured value represented as:

$$MABE=\frac{1}{N}\sum_{i=1}^{N}\left|\left(V_{i,P}-V_{i,M}\right)\right|$$ (31)

The MAPE is the mean absolute percentage difference between the predicted and measured wind power density represented:

$$MAPE=\frac{1}{N}\sum_{i=1}^{N}|\left(\frac{V_{i,P}-V_{i,M}}{V_{i,M}}\right)|\times 100$$ (32)

The RMSE presents the accuracy of the model by comparing the deviation between predicted and measured wind power density. The value of RMSE is always positive and it is defined as:

$$RMSE=\sqrt{\frac{1}{N}\sum_{i=1}^{N}{\left(V_{i,P}-V_{i,M}\right)}^{2}}$$ (33)

The coefficient of determination (R^2^) indicates the strength of linear relationship between the predicted and measured wind power density. R^2^ is obtained by:

$$R^{2}=1-\frac{\sum_{i=1}^{N}{\left(V_{i,P}-V_{i,M}\right)}^{2}}{\sum_{i=1}^{x}{\left(V_{i,M}-V_{M,avg}\right)}^{2}}$$ (34)

In the Eqs (31–34), *V*_*i*,*P*_ and *V*_*i*,*M*_ are wind power density estimated from developed prediction models and measured data respectively.

## Result and discussion

### Monthly wind power density prediction

**Fig 6:**
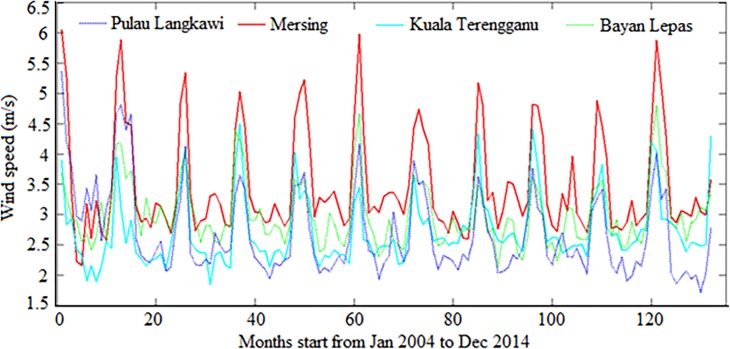
Visual presentation of wind speed at 50m hub height of underlying locations.

In the current study, hybrid ANFIS (ANFIS-PSO, ANFIS-GA, ANFIS-DE) and standalone ANFIS models were developed to predict wind power density. The above-mentioned models were trained and tested with different data size. Tables 5 and 6 shows descriptive values of input and output parameters respectively such as; maximum (Max.), minimum (Min.), standard deviation (St Dev.) and range for different locations. On the other hand, Fig 6 shows visual presentation of wind speed at 50m hub height for study locations. It can be found in Fig 6 that maximum wind speed prevails in Mersing and followed by Pulau Langkawi, Bayan Lepas, and Kuala Terengganu. The raw data used in this study is presented in S1 Dataset: Supporting Information.rar.

**Table 5:** Descriptive statistical parameters of input (wind speed, m/s) for different locations.

Parameters	Locations			
	Mersing	Kuala Terengganu	Pulau Langkawi	Bayan Lepas
Max.	6.05	4.50	5.35	4.80
Min.	2.15	1.84	1.72	2.12
St dev.	0.87	0.57	0.72	0.52
Range	3.91	2.66	3.63	2.68

**Table 6:** Descriptive statistical parameters of measured WPD (W/m^2^) for different locations.

Parameters	Locations			
	Mersing	Kuala Terengganu	Pulau Langkawi	Bayan Lepas
Max.	152.85	90.35	104.50	78.31
Min.	6.44	4.49	4.07	6.66
St dev.	33.80	17.75	17.28	13.73
Range	146.41	100.43	71.66	85.86

Table 7 presents the error metrics, including MAPE, MABE, RMSE obtained while training the prediction models with the input-output data set for Mersing, Langkawi, Bayan Lepas and Kuala Terengganu. The data size for training and testing the models were P = 105 and Q = 27 respectively. In this study, the performance of the prediction models is categorized based on lowest RMSE. It can be observed from Table 7 that ANFIS-PSO model has lowest RMSE, MAPE and MABE in the training stage for the data set of Mersing, Bayan Lepas, and Kuala Terengganu whereas, ANFIS-GA ranks in the first for the data set of Pulau Langkawi. Table 8 summarizes the RMSE, MAPE and MABE performance metrics when testing data set of different underlying locations is applied to the predictions models. As can be seen from Table 8, the ANFIS-GA model ranks in the first for the underlying data sets of Mersing and Kuala Terengganu. On the other hand, ANFIS-PSO model provides the smallest error metrics for the data set of Pulau Langkawi and Bayan Lepas.

**Table 7:** A Statistical model comparison in training the models when P = 105 and Q = 27.

Locations	Rank	Prediction Models	Training			
			RMSE	MAPE (%)	MABE	R^2^
Mersing	1	ANFIS-PSO	4.96	5.14	2.61	0.9781
	2	ANFIS-GA	5.41	5.77	2.63	0.9756
	3	ANFIS-DE	5.50	9.70	3.03	0.9749
	4	ANFIS	5.82	10.88	3.07	0.9728
Pulau Langkawi	1	ANFIS-GA	3.13	10.17	1.89	0.9709
	2	ANFIS-PSO	3.16	10.96	1.90	0.9703
	3	ANFIS	3.26	11.70	1.96	0.9684
	4	ANFIS-DE	3.30	10.10	1.90	0.9676
Bayan Lepas	1	ANFIS-PSO	2.14	7.88	1.65	0.9762
	2	ANFIS	2.28	9.06	1.74	0.9709
	3	ANFIS-GA	2.34	8.52	1.72	0.9692
	4	ANFIS-DE	2.54	9.04	1.83	0.9638
Kuala Terengganu	1	ANFIS-PSO	3.73	8.90	1.91	0.9523
	2	ANFIS-GA	4.0	10.30	2.19	0.9449
	3	ANFIS	4.03	14.57	2.29	0.9448
	4	ANFIS-DE	4.37	18.30	2.88	0.9392

**Table 8:** A Statistical model comparison in testing the models when P = 105 and Q = 27.

Locations	Rank	Prediction models	Testing			
			RMSE	MAPE (%)	MABE	R^2^
Mersing	1	ANFIS-GA	5.04	5.25	2.52	0.9701
	2	ANFIS-PSO	5.10	5.88	2.67	0.9691
	3	ANFIS	5.14	6.65	2.74	0.9690
	4	ANFIS-DE	5.56	9.22	3.26	0.9636
Pulau Langkawi	1	ANFIS-PSO	1.53	11.11	1.21	0.9774
	2	ANFIS-DE	1.59	13.07	1.19	0.9775
	3	ANFIS-GA	1.72	10.27	1.23	0.9737
	4	ANFIS	2.17	23.07	1.79	0.9578
Bayan Lepas	1	ANFIS-PSO	2.37	8.17	1.82	0.9749
	2	ANFIS	2.49	9.02	1.92	0.9720
	3	ANFIS-GA	2.75	7.27	1.83	0.9659
	4	ANFIS-DE	2.81	8.84	1.96	0.9643
Kuala Terengganu	1	ANFIS-GA	4.79	13.65	3.06	0.9412
	2	ANFIS-PSO	4.92	10.77	3.54	0.9456
	3	ANFIS-DE	5.01	21.67	3.70	0.9355
	4	ANFIS	5.37	12.97	3.15	0.9261

It is important to mention that sometimes ANFIS model provides better performance than hybrid ANFIS models but does not provide the best model performance for any of the underlying locations. For instance, ANFIS shows second and third best performance for the data set of Bayan Lepas and Mersing respectively when testing the prediction models.

The R^2^ is the correlation between measured and predicted WPD, which has the highest value of one. A pronounced observation of R^2^ from Tables 7 and 8 revealed a very good correlation between measured and predicted WPD obtained when training and testing the prediction models using the data set from Mersing, Pulau Langkawi, and Bayan Lepas. On the other hand, the measured and predicted WPD of Kuala Terengganu suffers comparatively lower correlation when training and testing the prediction models.

Furthermore, the performance of the developed prediction models is compared to different training and testing data size to illustrate the effect of data size on the prediction accuracy. Tables (9–12) summarize the RMSE, MABE, MAPE, and R^2^ when input-output data sets of Mersing, Pulau Langkawi, Bayan Lepas and Kuala Terengganu respectively are applied to the prediction models. In this case, training and testing dataset consists of P = 92 (70%) and Q = 40 (30%) observations during the period January 2004 to August 2011 and September 2011 to December 2014 respectively. Furthermore, in order to choose the best data size that will provide optimal error in WPD prediction, all the developed models were trained and tested with another input-output data size, which consists of P = 80 (60%) and Q = 52 (40%) observations during the period January 2004 to August 2010 and September 2010 to December 2014 respectively. The statistical error metrics (RMSE, MABE, MAPE, and R^2^) for this category of training and testing data set are also shown in Tables (9–12).

**Table 9:** Effect of data size for the prediction wind power density of Mersing.

Data Size	Training						Testing					
	Rank	Prediction models	RMSE	MABE	MAPE (%)	R^2^	Rank	Prediction models	RMSE (W)	MABE (W)	MAPE (%)	R^2^
P = 92, Q = 40	1	ANFIS-PSO	5.23	2.64	6.88	0.9786	1	ANFIS-PSO	5.37	2.75	5.95	0.9663
	2	ANFIS-GA	5.36	2.92	9.45	0.9712	2	ANFIS-GA	5.46	2.89	6.53	0.9651
	3	ANFIS	5.39	2.92	10.28	0.9767	3	ANFIS	5.55	2.94	7.06	0.9640
	4	ANFIS-DE	5.52	2.81	7.84	0.9756	4	ANFIS-DE	5.72	2.84	5.68	0.9617
P = 80, Q = 52	1	ANFIS-PSO	5.41	2.82	6.40	0.9780	1	ANFIS-PSO	5.22	2.79	6.40	0.9662
	2	ANFIS-GA	5.59	3.33	8.95	0.9765	2	ANFIS-DE	5.33	3.58	11.56	0.9652
	3	ANFIS	5.82	3.13	12.46	0.9745	3	ANFIS-GA	5.46	3.34	8.95	0.9634
	4	ANFIS-DE	6.21	4.29	11.56	0.9709	4	ANFIS	5.48	3.12	7.55	0.9632

**Table 10:** Effect of data size for the prediction wind power density of Bayan Lepas.

Data Size	Training						Testing					
	Rank	Prediction models	RMSE	MABE	MAPE (%)	R^2^	Rank	Prediction models	RMSE (W)	MABE (W)	MAPE (%)	R^2^
P = 92, Q = 40	1	ANFIS-PSO	2.17	1.64	7.85	0.9748	1	ANFIS-PSO	2.54	1.74	7.83	0.9684
	2	ANFIS-GA	2.22	1.69	7.99	0.9740	2	ANFIS-GA	2.62	1.78	7.67	0.9584
	3	ANFIS	2.24	1.72	8.95	0.9698	3	ANFIS	2.73	1.93	9.95	0.9541
	4	ANFIS-DE	2.38	1.73	7.98	0.9738	4	ANFIS-DE	2.80	1.87	8.25	0.9577
P = 80, Q = 52	1	ANFIS-PSO	2.18	1.56	7.29	0.9770	1	ANFIS	2.65	1.96	11.67	0.9441
	2	ANFIS	2.25	1.73	8.35	0.9756	2	ANFIS-PSO	2.74	1.98	11.14	0.9409
	3	ANFIS-GA	2.46	1.77	7.71	0.9707	3	ANFIS-DE	2.77	2.18	11.76	0.9398
	4	ANFIS-DE	3.34	2.34	10.23	0.9461	4	ANFIS-GA	3.38	2.0	9.39	0.9252

**Table 11:** Effect of data size for the prediction wind power density of Pulau Langkawi.

Data Size	Training						Testing					
	Rank	Prediction models	RMSE	MABE	MAPE (%)	R^2^	Rank	Prediction models	RMSE (W)	MABE (W)	MAPE (%)	R^2^
P = 92, Q = 40	1	ANFIS-PSO	3.29	1.92	10.28	0.9707	1	ANFIS-PSO	1.69	1.38	12.28	0.9717
	2	ANFIS-GA	3.34	1.92	10.09	0.9698	2	ANFIS-GA	1.71	1.31	11.89	0.9707
	3	ANFIS	3.38	2.03	11.60	0.9690	3	ANFIS-DE	1.88	1.44	11.55	0.9650
	4	ANFIS-DE	3.43	2.21	13.08	0.9682	4	ANFIS	2.23	1.84	20.39	0.9508
P = 80, Q = 52	1	ANFIS	3.45	2.14	11.64	0.9705	1	ANFIS-GA	2.04	1.64	18.18	0.9572
	2	ANFIS-GA	3.58	2.19	12.61	0.9668	2	ANFIS	2.20	1.66	17.19	0.9501
	3	ANFIS-DE	4.06	3.04	19.24	0.9591	3	ANFIS-PSO	2.37	1.68	13.91	0.9421
	4	ANFIS-PSO	4.15	2.55	11.60	0.9572	4	ANFIS-DE	3.23	2.68	26.17	0.8921

**Table 12:** Effect of data size for the prediction wind power density of Kaula Terengganu.

Data Size	Training						Testing					
	Rank	Prediction models	RMSE	MABE	MAPE (%)	R^2^	Rank	Prediction models	RMSE (W)	MABE (W)	MAPE (%)	R^2^
P = 92, Q = 40	1	ANFIS	4.07	2.28	12.61	0.9407	1	ANFIS-PSO	4.15	2.72	12.74	0.9436
	2	ANFIS-PSO	4.15	2.32	11.61	0.9385	2	ANFIS-DE	4.24	2.50	13.07	0.9531
	3	ANFIS-GA	4.32	2.33	12.40	0.9421	3	ANFIS	4.61	2.68	11.83	0.9448
	4	ANFIS-DE	4.39	2.37	12.06	0.9307	4	ANFIS-GA	5.16	2.78	11.95	0.9307
P = 80, Q = 52	1	ANFIS-PSO	3.99	2.05	10.06	0.9438	1	ANFIS-PSO	4.58	2.85	12.43	0.9405
	2	ANFIS-GA	4.06	2.36	13.13	0.9415	2	ANFIS	4.73	3.27	16.18	0.9307
	3	ANFIS	4.11	2.23	12.85	0.9401	3	ANFIS-DE	4.92	3.74	24.15	0.9363
	4	ANFIS-DE	4.70	3.04	21.10	0.9217	4	ANFIS-GA	4.94	3.64	19.35	0.9312

A profound observation on Tables (7–12) reveals that:

- For WPD prediction of Mersing, ANFIS-PSO is the best model when training the models and the value of RMSE obtained are 4.96, 5.23 and 5.41 for the data size P = 105, Q = 27; P = 92, Q = 40, and P = 80, Q = 52 respectively. On the other hand, when testing the prediction models ANFIS-GA is the best for the data size P = 105, Q = 27 and having RMSE of 5.04 whereas, ANFIS-PSO is the best model when data size P = 92, Q = 40, and P = 80, Q = 52 resulting RMSE of 5.37 and 5.22 respectively.
- For WPD prediction of Bayan Lepas, ANFIS-PSO shows the best performance for all above data size when training the prediction models. In this case, the computed values of RMSE are 2.14, 2.17 and 2.18 when the training data sizes are of P = 105, Q = 27; P = 92, Q = 40, and P = 80, Q = 52 respectively. Then again, ANFIS-PSO shows best performance for a testing data size of P = 105, Q = 27 and P = 92, Q = 40 resulting RMSE of 2.37 and 2.54 respectively. It is not surprising that ANFIS model has the best accuracy when testing data size of P = 80, Q = 52 having RMSE of 2.65.
- For WPD prediction of Pulau Langkawi, ANFIS-GA and ANFIS-PSO show the best performance in training and testing the models respectively with data size of P = 105, Q = 27 which results in RMSE of 3.13 and 1.53 in training and testing respectively. In case of the data size P = 92 and Q = 40, ANFIS-PSO ranks in first both training and validation with RMSE of 3.29 and 1.69 respectively. For the training and testing data size P = 80, Q = 52; ANFIS and ANFIS-GA model show optimal RMSE, which are 3.45 and 2.04 in training and testing respectively.
- For WPD prediction of Kuala Terengganu, ANFIS-PSO and ANFIS-GA show the best performance in training and testing the models respectively with data size of P = 105, Q = 27 which results in RMSE of 3.73 and 4.79 respectively. For the data size P = 92, Q = 40; ANFIS model shows optimal RMSE, which is 3.45 in training stage and ANFIS-PSO presents optimal RMSE of 2.23 in the testing stage. In case of data size P = 80 and Q = 52, ANFIS-PSO ranks in first both training and validation of the prediction models with RMSE of 3.99 and 4.58 respectively.

Based on the above discussion, it is clear that P = 105, Q = 27 data size applied to the prediction models provides minimal error for WPD prediction of all underlying locations. For this data size, overall ANFIS-PSO and ANFIS-GA showed a higher correlation between measured and predicted WPD all locations both in training and testing stages.

On the other hand, when P = 92, Q = 40 data set are applied to the prediction models, ANFIS-PSO shows the best performance in both training and testing stages for predicting WPD of Mersing, Pulau Langkawi, and Bayan Lepas. However, when predicting WPD of Kuala Terengganu, ANFIS-PSO ranks second in training and first in the testing stage. It is important to mention that P = 80, Q = 52 data size shows the largest error for predicting WPD of all underlying locations, and therefore, we do not want to use P = 80, Q = 52 data set for model performance justification.

**Fig 7:**
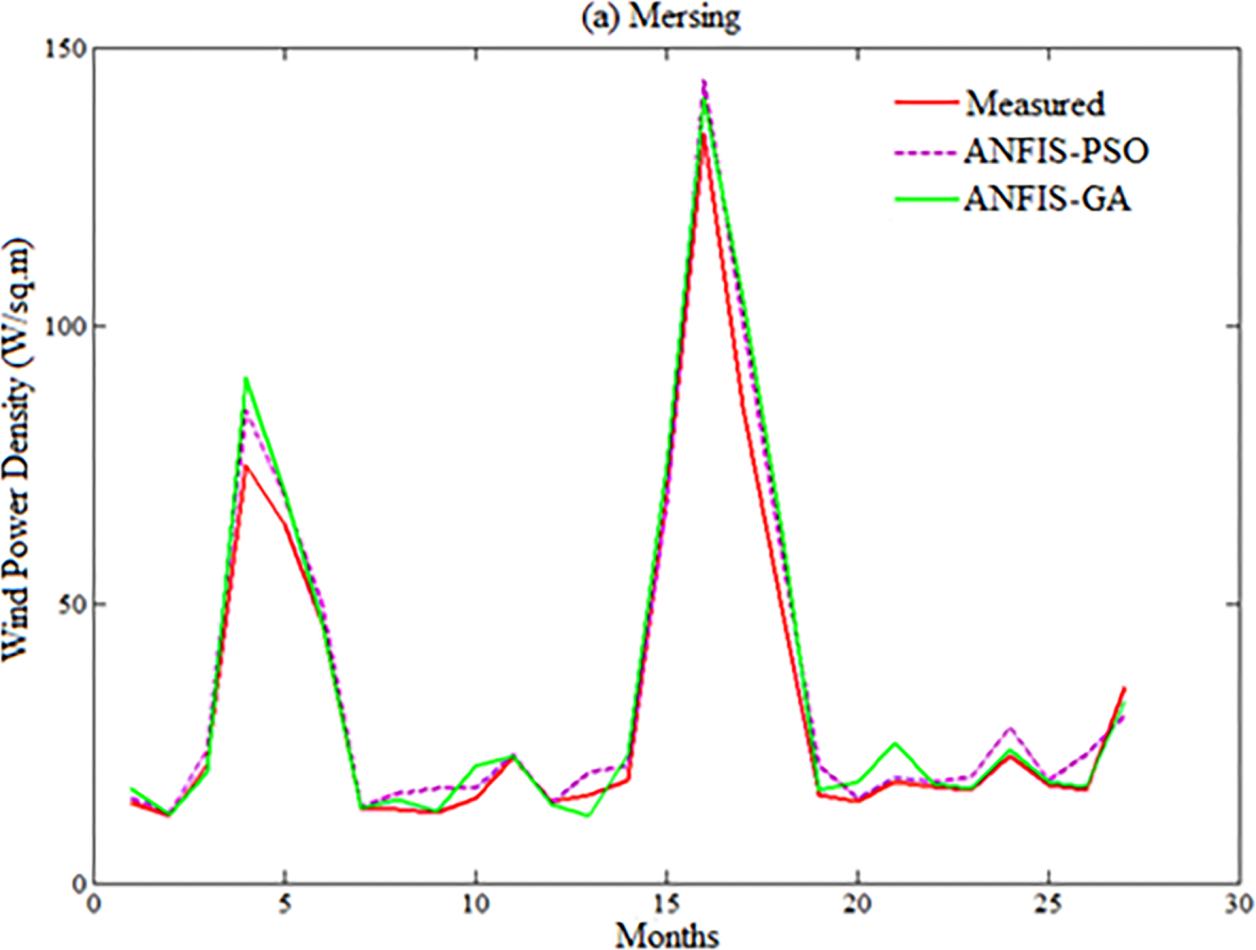
Comparison of WPD prediction from proposed methods when testing with measured WPD value for Mersing.

**Fig 8:**
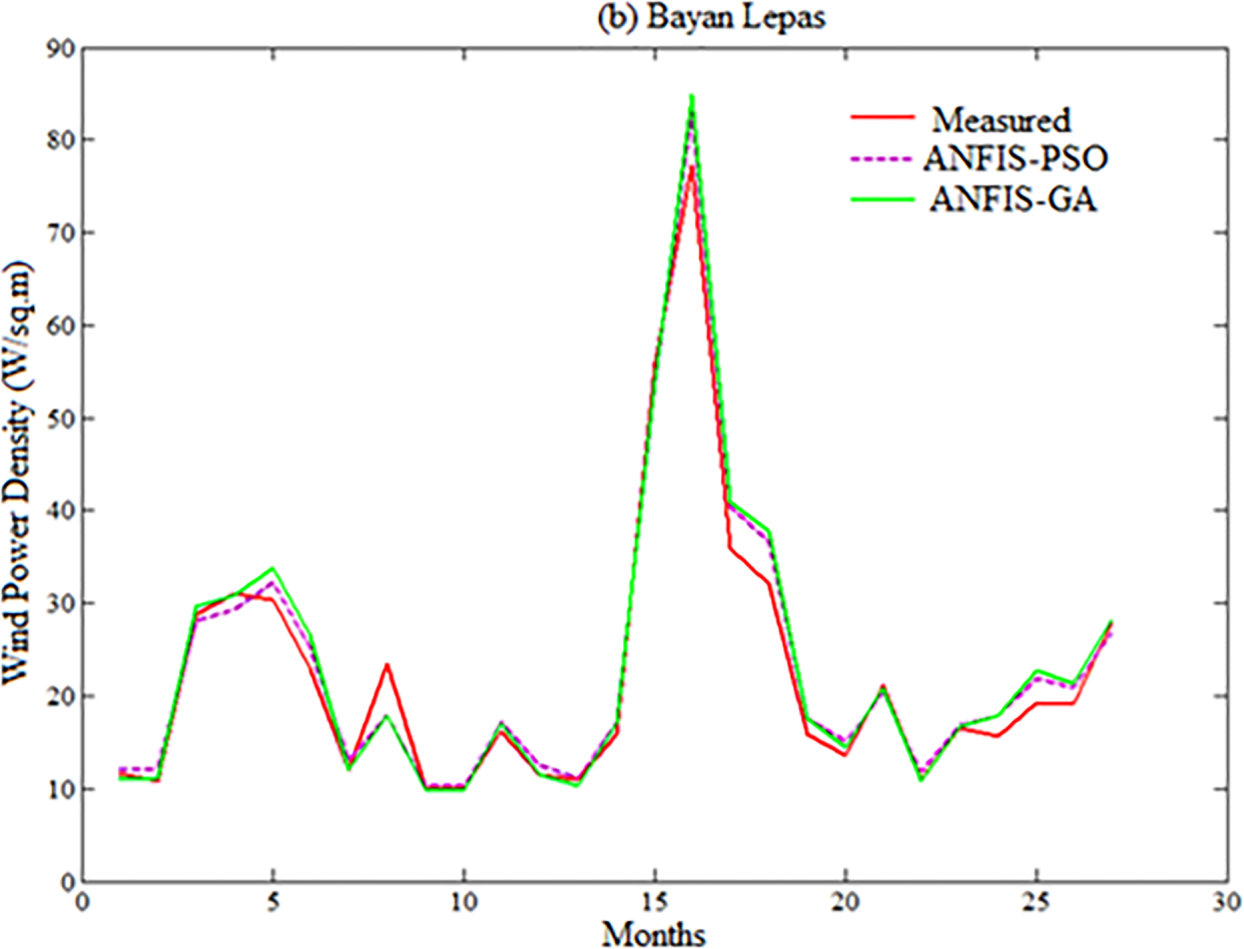
Comparison of WPD prediction from proposed methods when testing with measured WPD value for Bayan Lepas.

**Fig 9:**
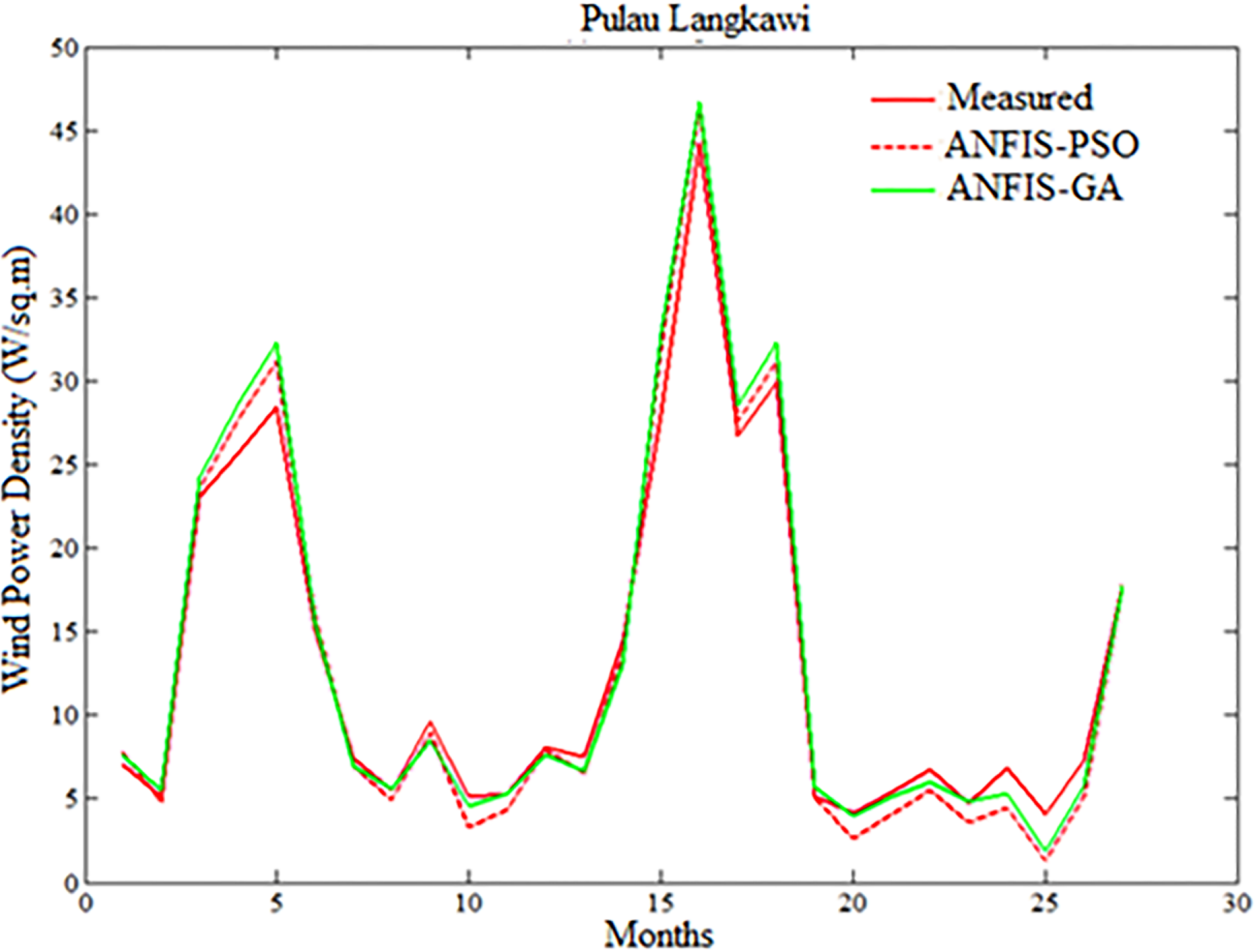
Comparison of WPD prediction of the proposed method with measured value for Pulau Langkawi.

**Fig 10:**
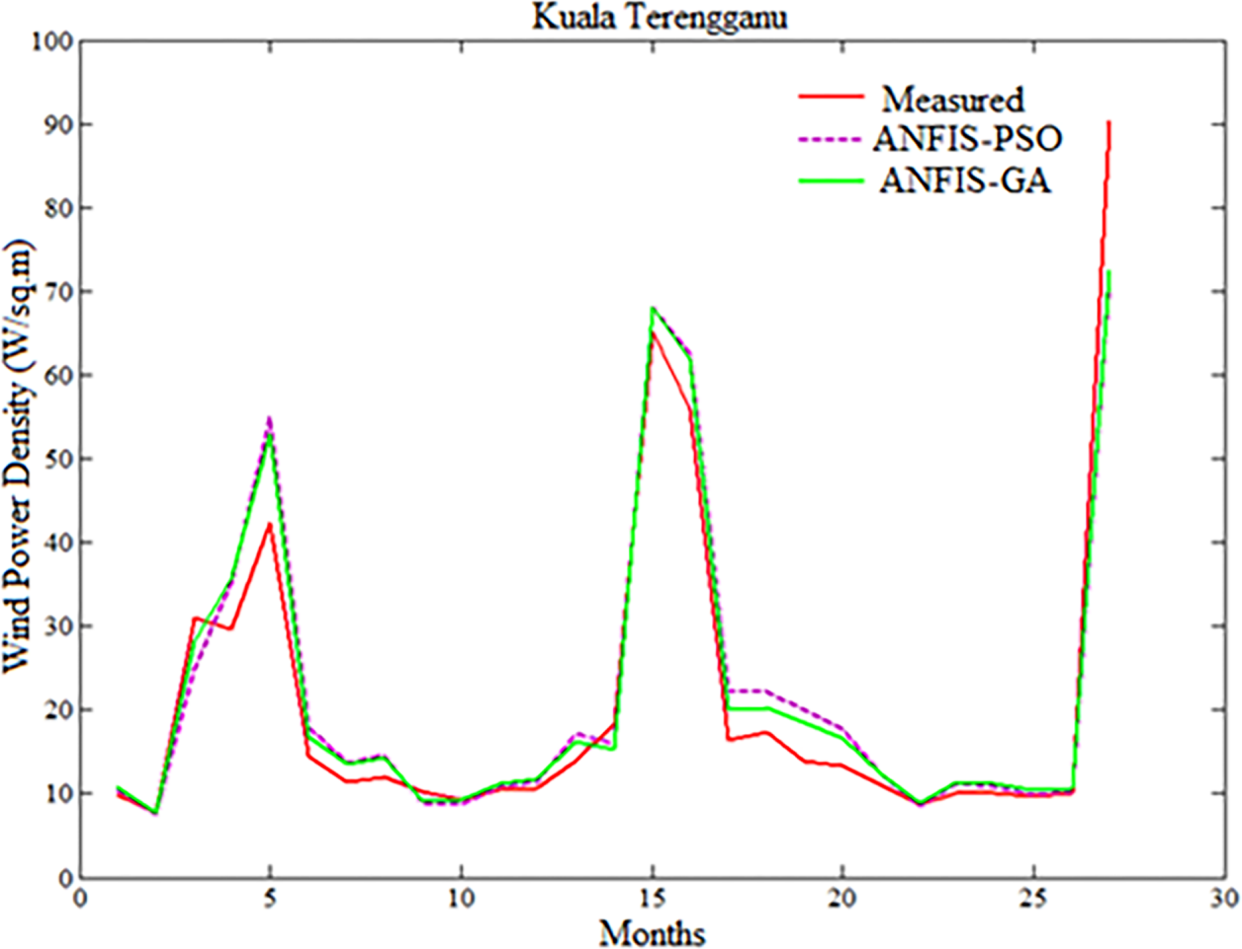
Comparison of WPD prediction of proposed methods with measured value for Kuala Terengganu.

Figs 7–10 depicts the visual presentation of measured and predicted WPD when testing using ANFIS-PSO and ANFIS-GA for Mersing, Bayan Lepas, Pulau Langkawi and Kuala Terengganu respectively with best data size i.e. P = 105, Q = 27.

**Fig 11:**
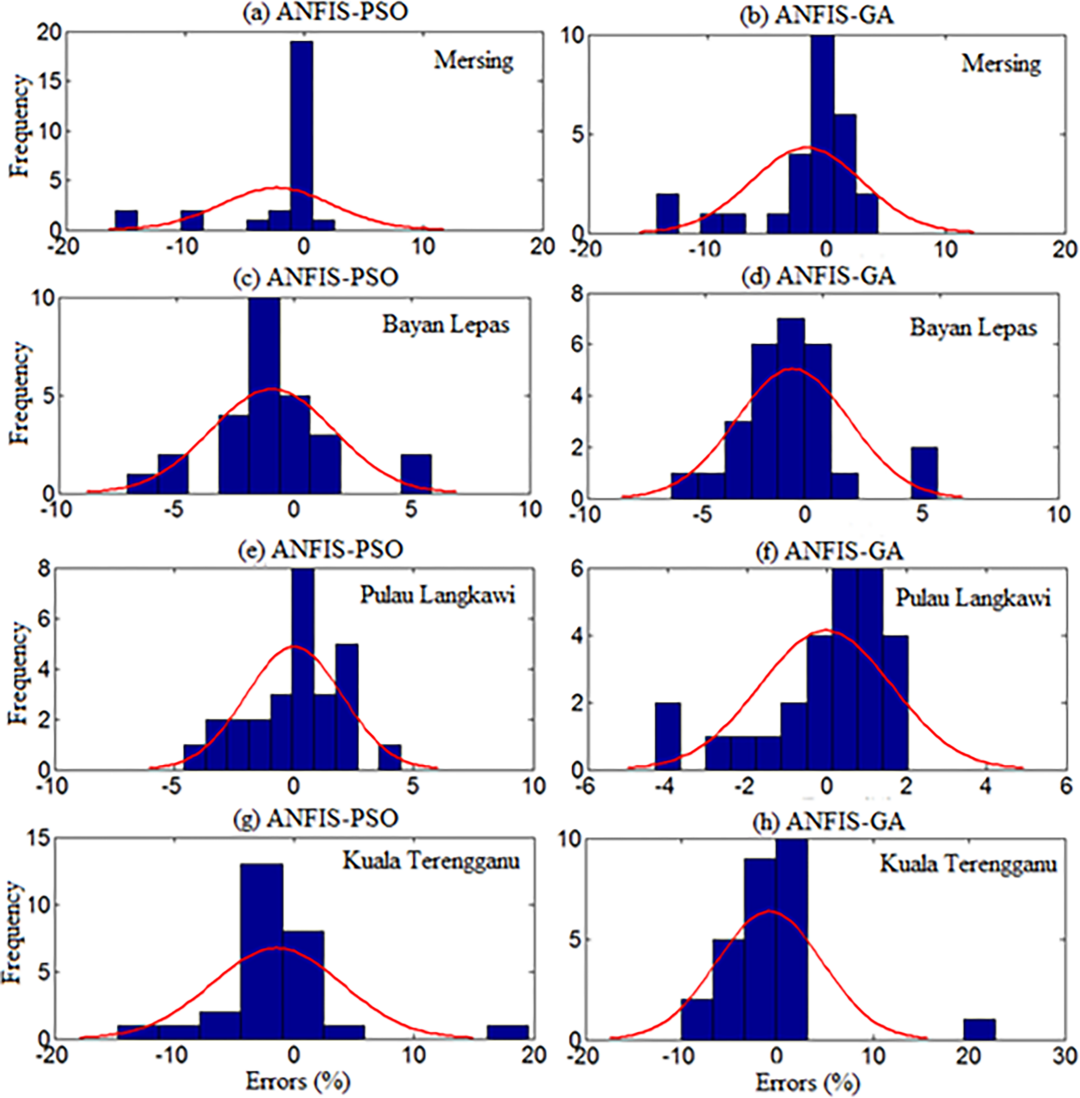
Error distribution when testing ANFIS-PSO and ANFIS-GA for (a, b) Mersing, (c, d) Bayan Lepas (e, f) Pulau Langkawi, (g, h) Kuala Terengganu.

However, both ANFIS-PSO and ANFIS-GA seem to be able to provide an overall good performance both in training and testing stages. Therefore, hybrid ANFIS, especially ANFIS-PSO and ANFIS-GA can be suggested for practical utilization in WPD prediction for the locations having similar wind resource conditions. The Fig 11 shows the error distribution of WPD prediction using ANFIS-PSO and ANFIS-GA for underlying locations. According to the definition of relative percentage error (RPE) presented in [27, 28], the RPE falls in an interval of -10% to 10% can be considered acceptable. The computed value of RPE presented in the Fig 11 is obtained from the proposed ANFIS-PSO and ANFIS-GA when 27 months testing data set of underlying locations applied to the models. It can be observed that most of the wind power values obtained via the proposed ANFIS-PSO and ANFIS-GA model fall within the range of -5% up to 5%. In case of Mersing and Kuala Terengganu, only one prediction RPE falls outside (-10% to 10%) range for both ANFIS-PSO and ANFIS-GA.

**Fig 12:**
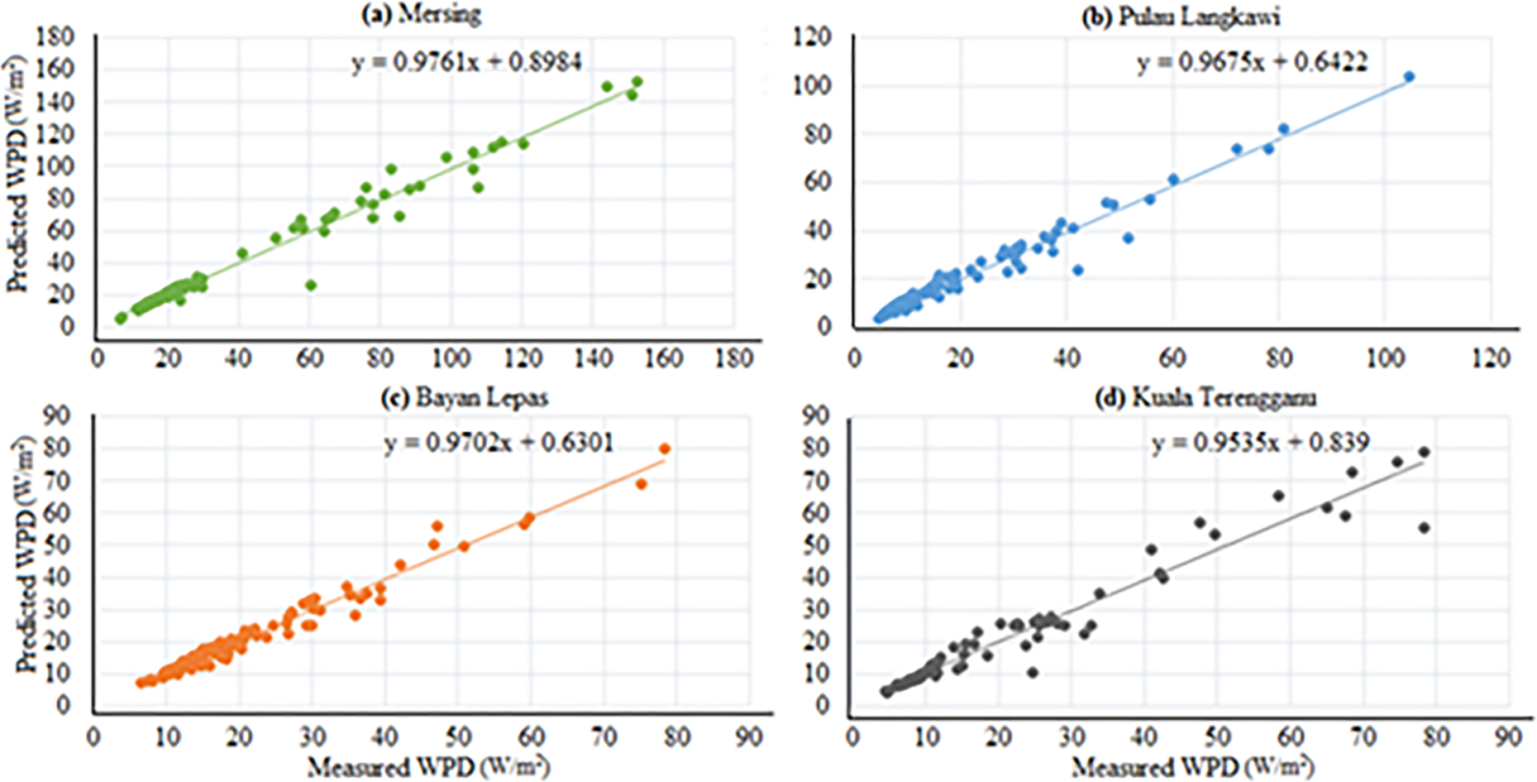
Measured versus predicted WPD when training ANFIS-PSO model (a) Mersing, (b) Pulau Langkawi, (c) Bayan Lepas and (d) Kuala Terengganu.

**Fig 13:**
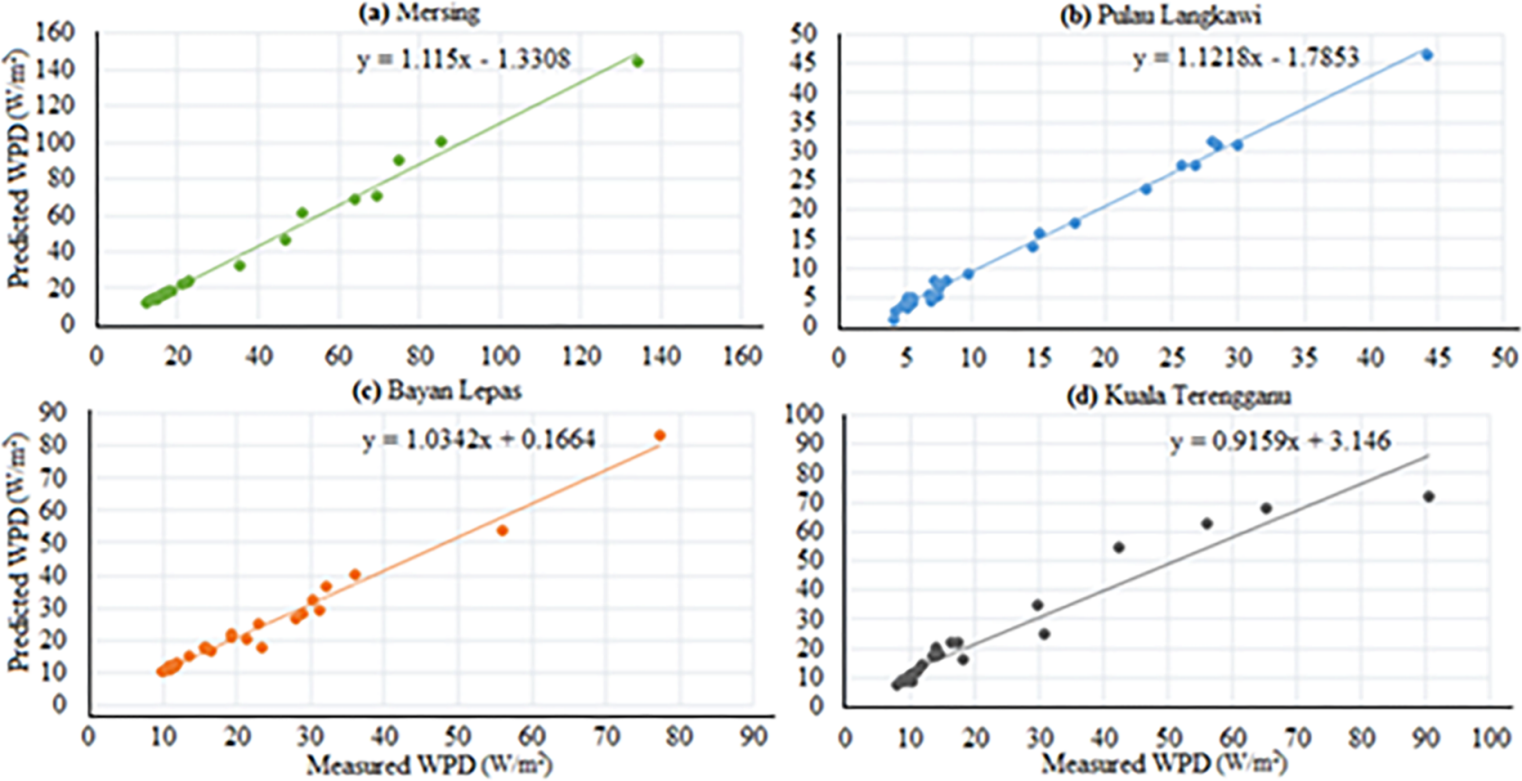
Measured versus predicted WPD when testing ANFIS-PSO model (a) Mersing, (b) Pulau Langkawi, (c) Bayan Lepas and (d) Kuala Terengganu.

Based on the above discussion, the performance of ANFIS-PSO can be considered the best among other the models. The Figs 12 and 13 show the visual presentation of regression analysis of measured and predicted WPD computed from ANFIS-PSO for all underlying locations when P = 105, Q = 27. The Fig 12 shows the correlation between actual and predicted WPD obtained from ANFIS-PSO method. The correlation coefficient (R^2^) is the agreement between measured and predicted values, which has the highest value of one. The R^2^ obtained when training proposed the ANFIS-PSO model are 0.9781, 0.9703, 0.9762 and 0.9523 for Mersing, Pulau Langkawi, Bayan Lepas and Kuala Terengganu respectively, which indicates a very high and adequate prediction performance of ANFIS-PSO model.

Again, the linear regression analysis presented in Fig 13 shows the correlation between actual and predicted WPD obtained from ANFIS-PSO when testing the prediction models with data from different underlying locations. The R^2^ obtained while testing the proposed ANFIS-PSO model for Mersing, Pulau Langkawi, Bayan Lepas and Kuala Terengganu are 0.9691, 0.9774, 0.9749 and 0.9456 respectively, which supports that ANFIS-PSO has a high precision for the prediction of WPD.

## Weekly wind power density prediction

**Fig 14:**
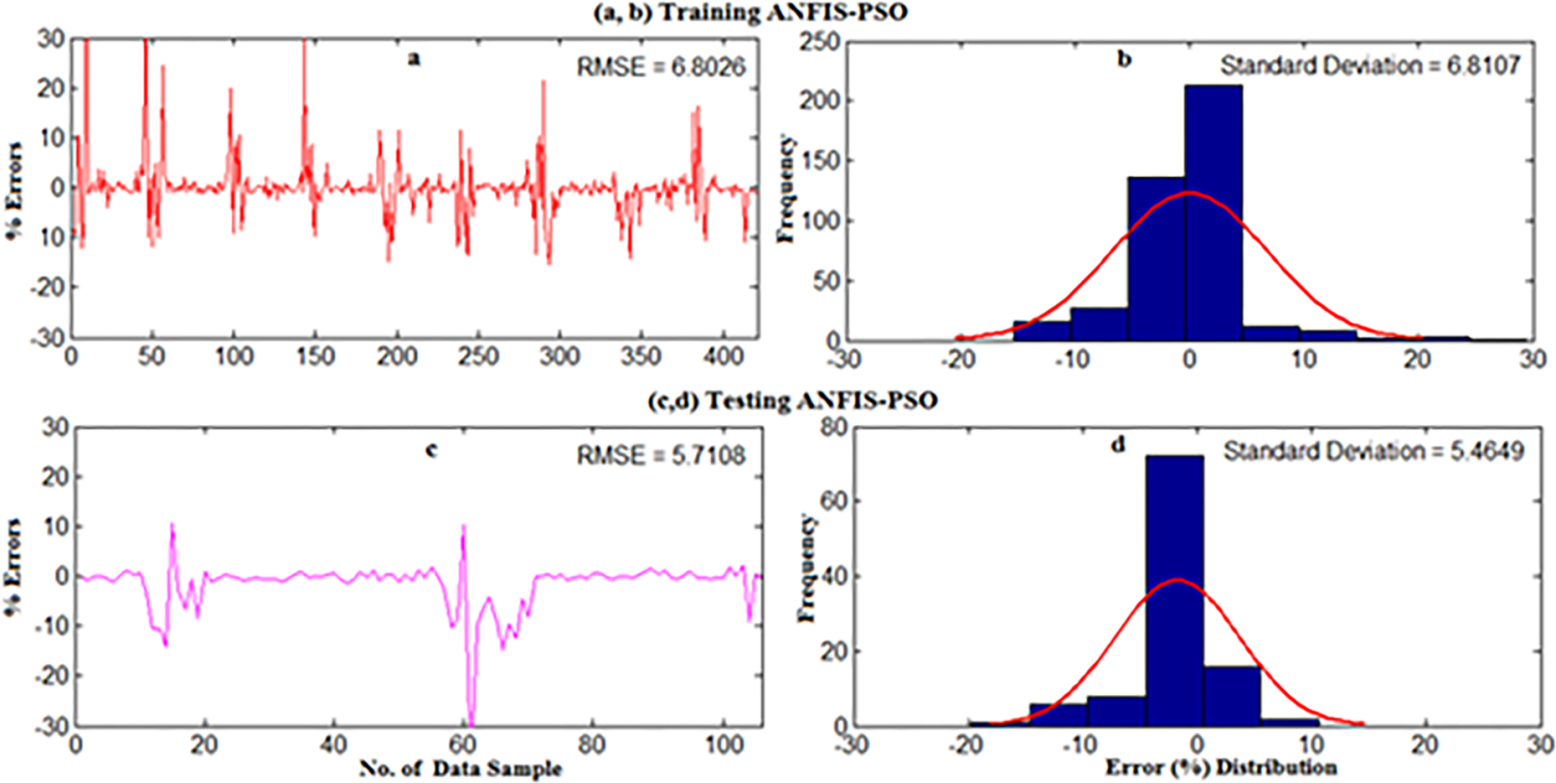
Error distribution when (a,b) Training and (c,d) Testing the ANFIS-PSO model for weekly WPD prediction of Mersing.

**Fig 15:**
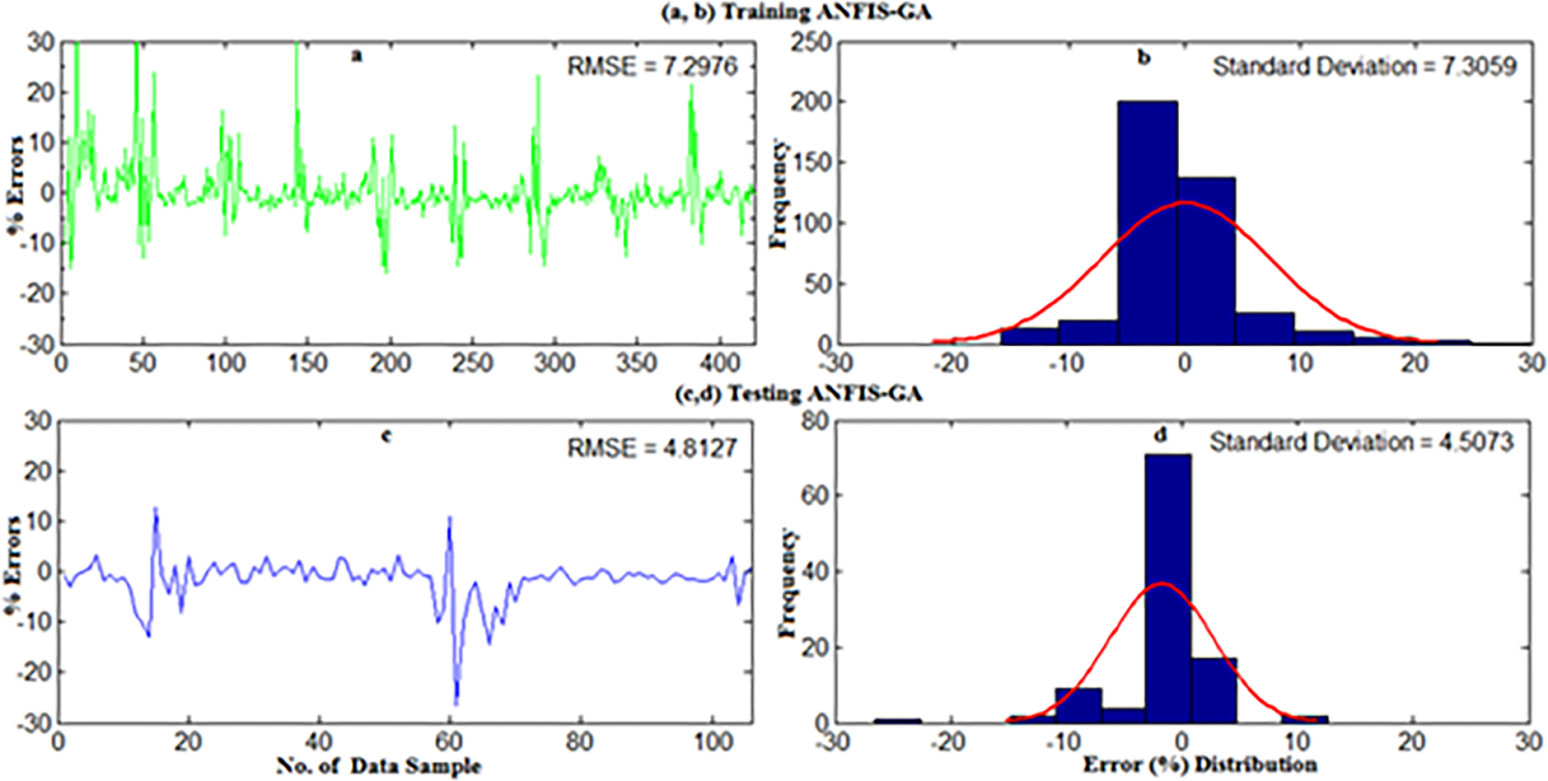
Error distribution when (a,b) Training and (c,d) Testing the ANFIS-GA model for weekly WPD prediction of Mersing.

**Fig 16:**
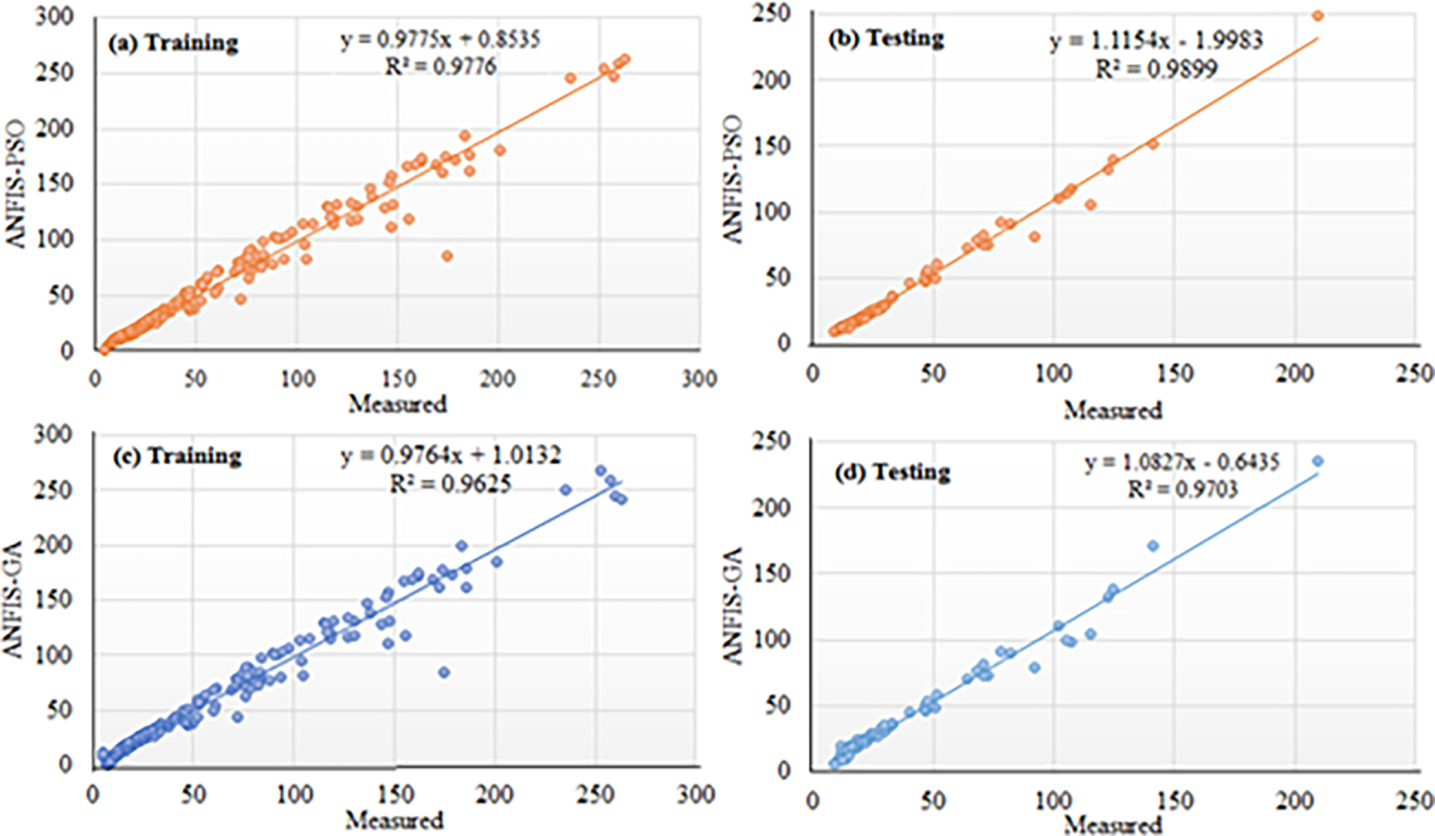
Regression plot of weekly WPD prediction for Mersing when training and testing the ANFIS-PSO and ANFIS-GA prediction model.

**Fig 17:**
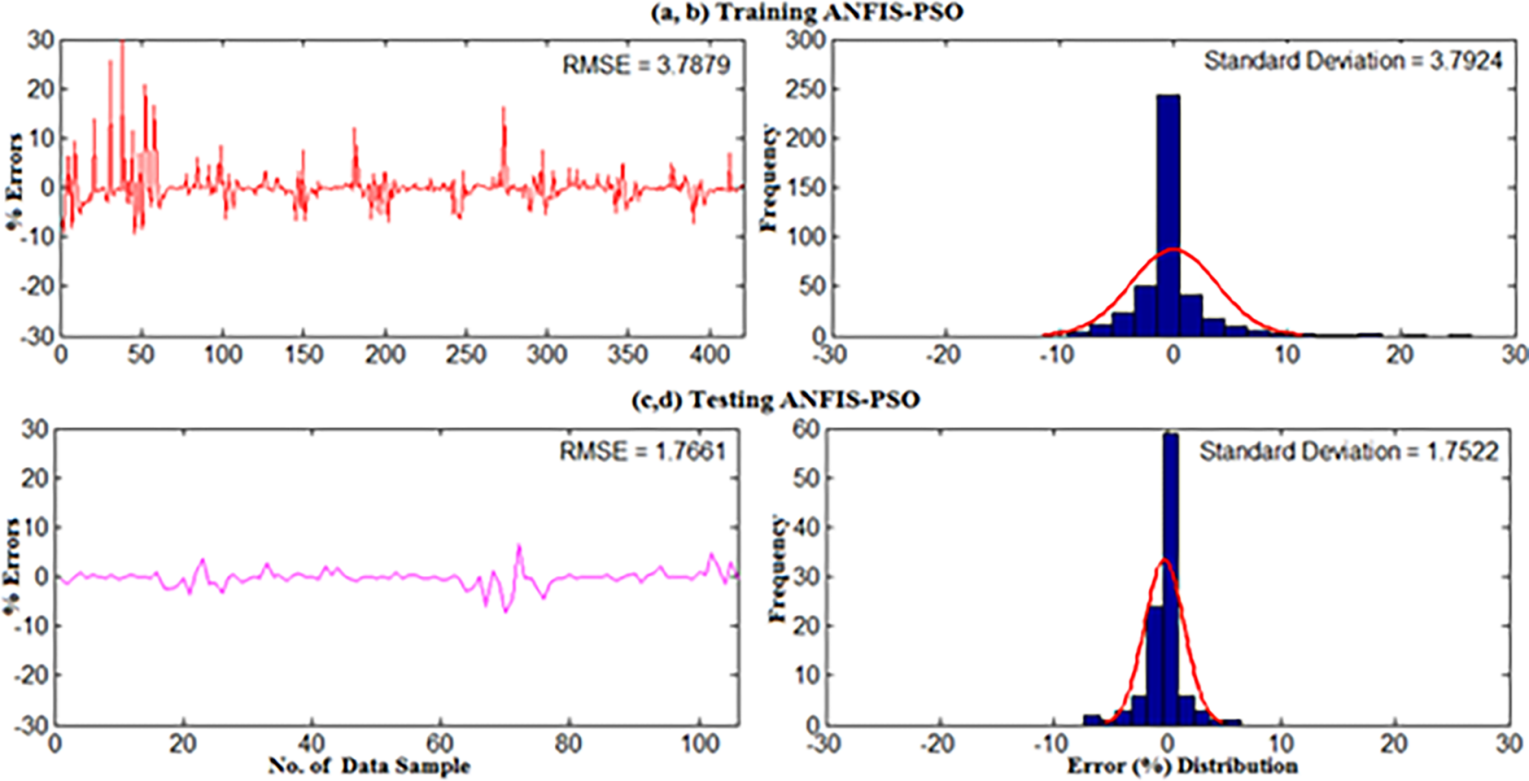
Error distribution when (a,b) Training and (c,d) Testing the ANFIS-PSO model for weekly WPD prediction of Pulau Langkawi.

**Fig 18:**
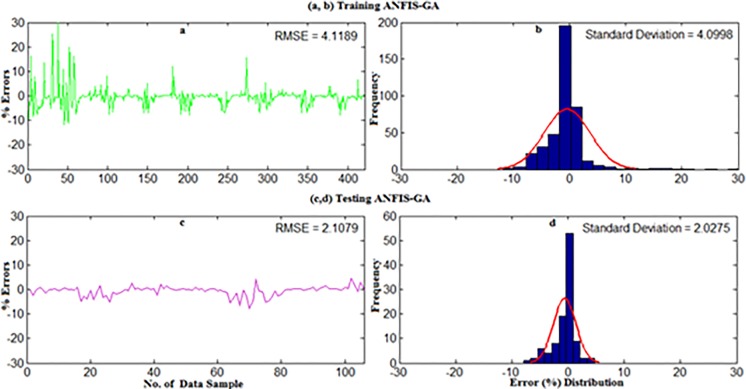
Error distribution when (a, b) Training and (c, d) Testing the ANFIS-GA model for weekly WPD prediction of Pulau Langkawi.

**Fig 19:**
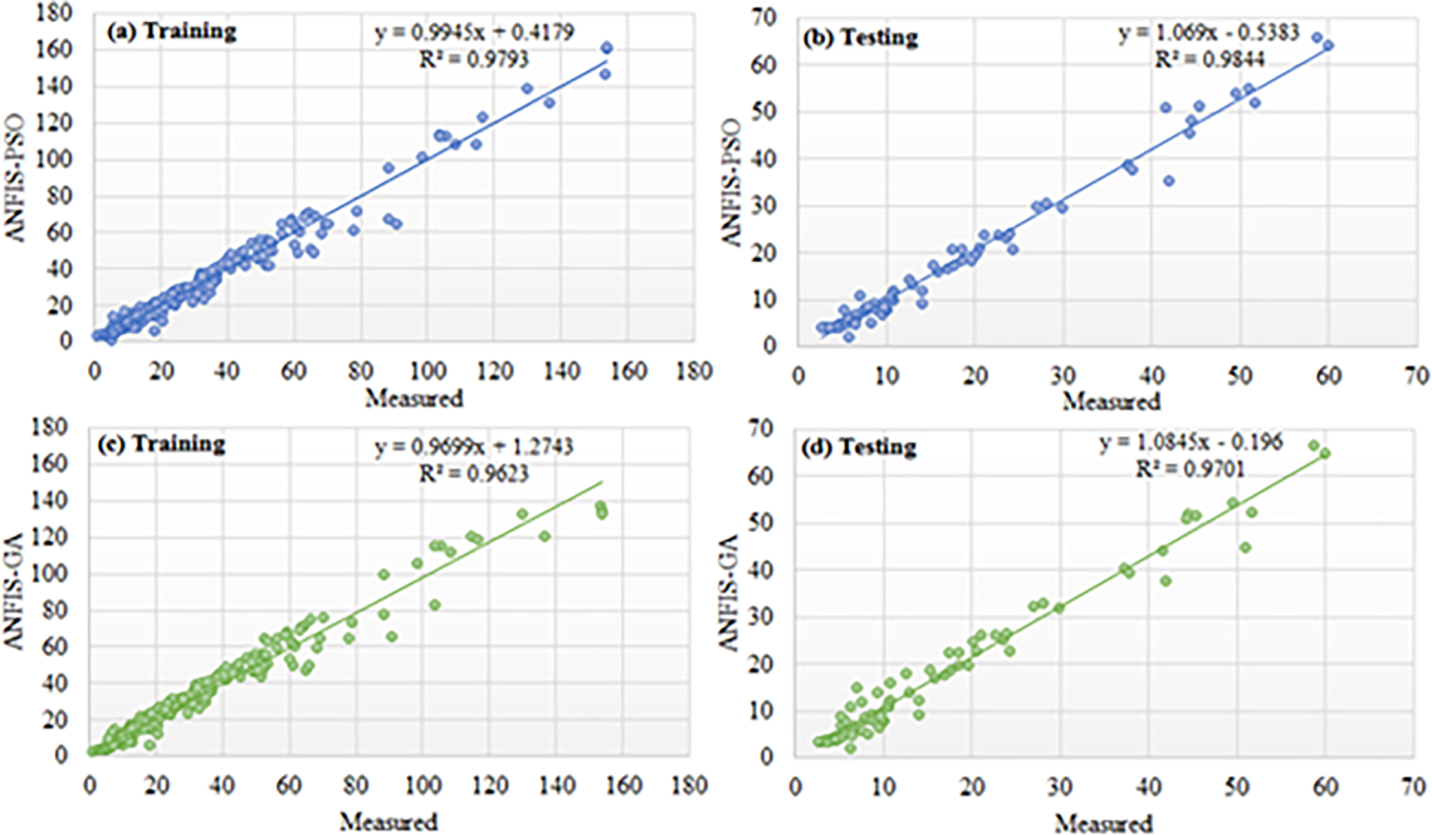
Regression plot of weekly WPD prediction for Pulau Langkawi when training and testing the ANFIS-PSO and ANFIS-GA prediction model.

The detailed explanation of data collection and site description can be found in section 2. For weekly wind power density prediction, 11 year’s (2004–2014) long-term daily average wind speed data were converted into the weekly average for all underlying locations in Malaysia namely Mersing, Kuala Terengganu, Pulau Langkawi and Bayan Lepas. Afterward, the weekly mean wind speed at 50m and the corresponding wind power density from measured data were applied on the developed standalone ANFIS and hybrid ANFIS models. The first 80% data and rest 20% data for training and testing were used respectively. The results presented in Figs (14–19) mainly obtained when training and testing the ANFIS-PSO and ANFIS-GA models as they were best prediction models for monthly wind power density prediction presented in section 5.1.

Figs 14 and 15 show the error distribution, RMSE, and MAPE whereas, Fig 16 presents regression analysis when training and testing data sets of Mersing were applied on ANFIS-PSO and ANFIS-GA prediction models. It can be observed from Figs 15 and 16 that most of the wind power values obtained via the proposed ANFIS-PSO and ANFIS-GA model fall within the range of -5% up to +5%. The RMSE were 5.72 and 4.82 when testing the ANFIS-PSO and ANFIS-GA respectively. It is mentioned in section 5.1 that the R^2^ is the correlation between measured and predicted WPD, which has the highest value of one. In Fig 17, the R^2^ were 0.9899 and 0.9703 when testing the ANFIS-PSO and ANFIS-GA respectively.

Similarly, Figs 17 and 18 show the error distribution, RMSE, and MAPE whereas, Fig 19 presents regression analysis when training and testing data sets of Langkawi were applied on ANFIS-PSO and ANFIS-GA prediction models. It can be observed from Figs 18 and 19 that most of the wind power values obtained via the proposed ANFIS-PSO and ANFIS-GA model fall within the range of -5% up to +5%. The RMSE were 1.76 and 2.11 when testing the ANFIS-PSO and ANFIS-GA respectively. It is mentioned in section 5.1 that the R^2^ is the correlation between measured and predicted WPD, which has the highest value of one. In Fig 19, the R^2^ were 0.9844 and 0.9701 when testing the ANFIS-PSO and ANFIS-GA respectively.

### Extrapolation capabilities of the proposed models

**Fig 20:**
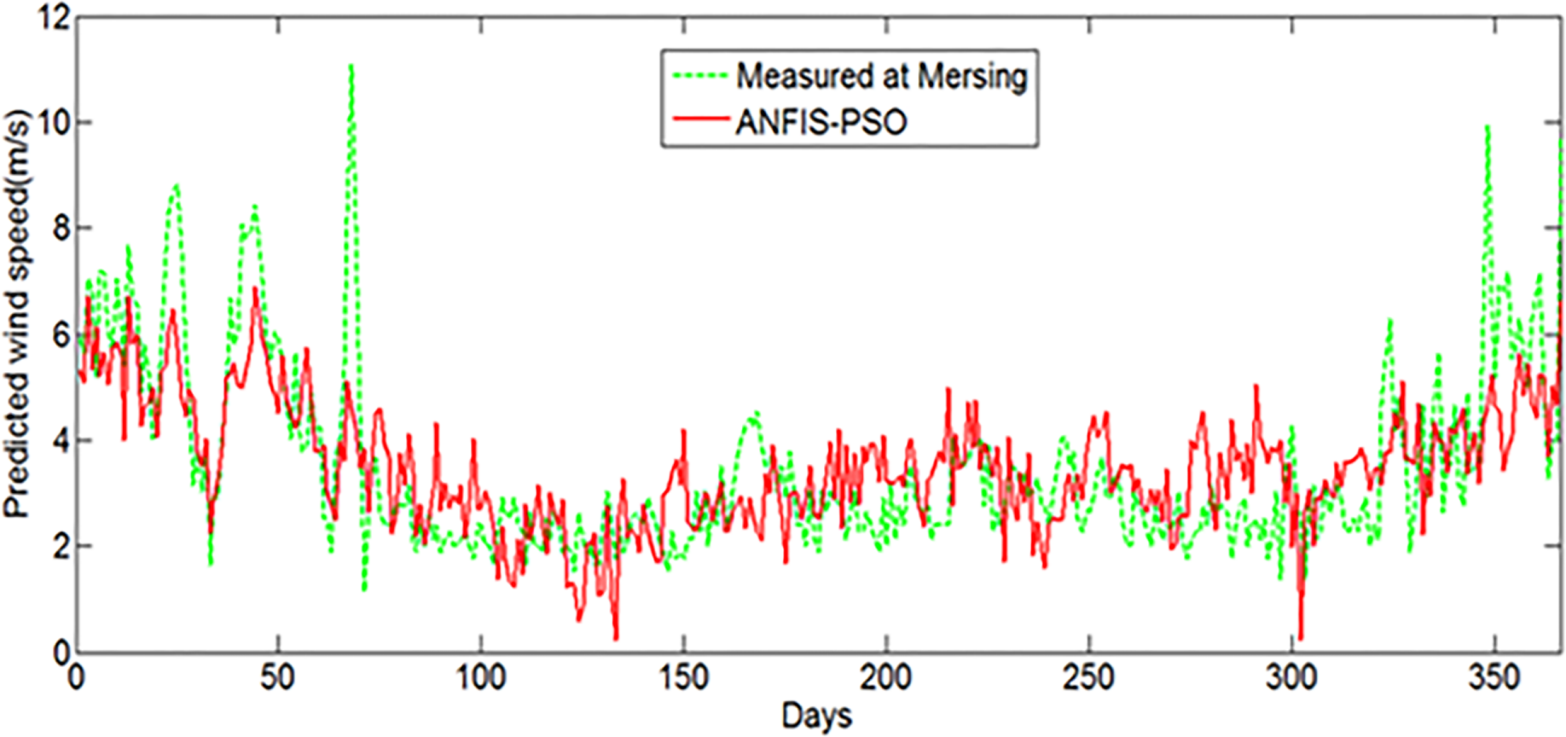
Extrapolation of daily average wind speed for Tioman Island using proposed ANFIS-PSO.

**Fig 21:**
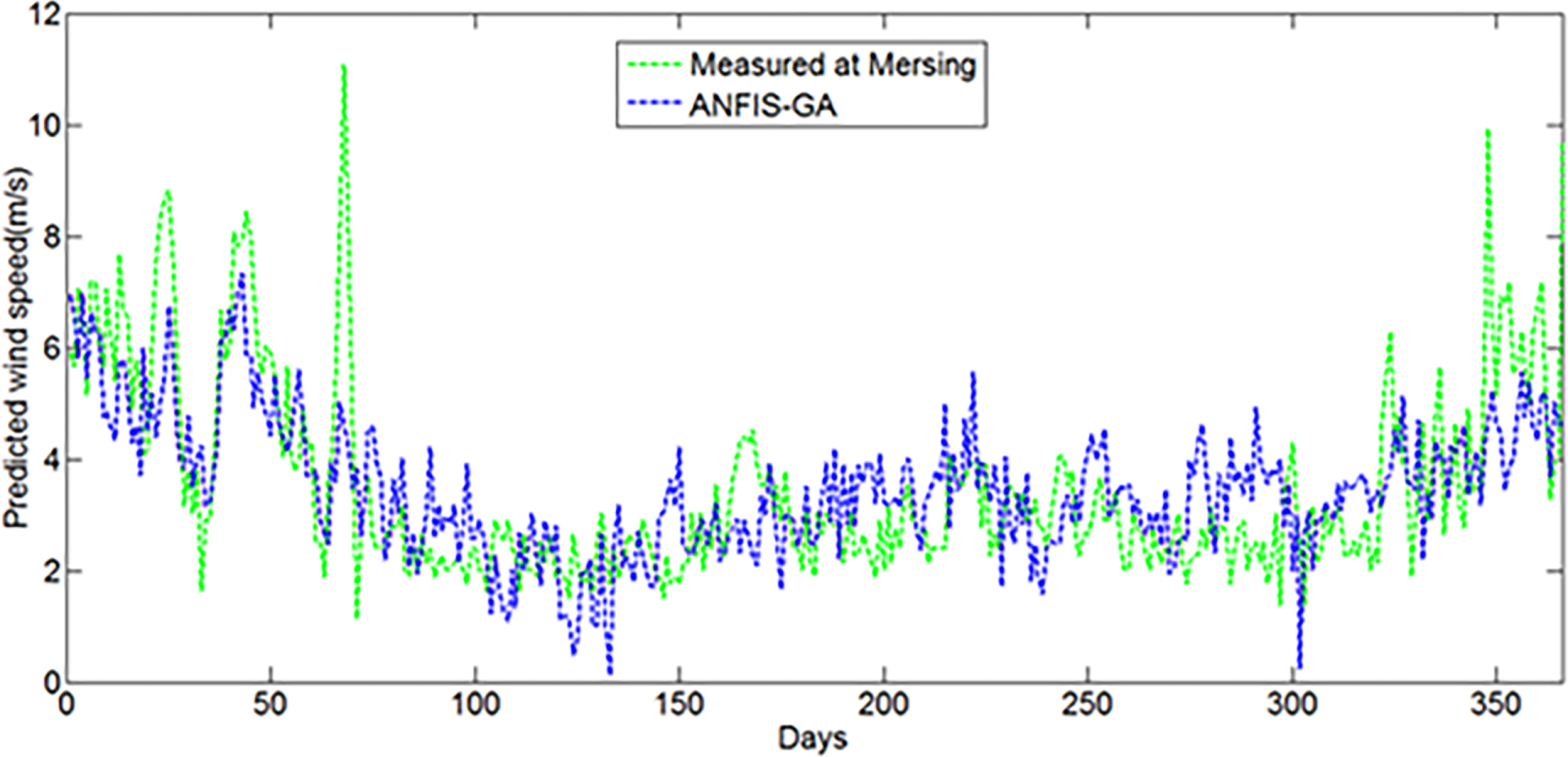
Extrapolation of daily average wind speed for Tioman Island using proposed ANFIS-GA.

Measured wind speed data are not available for many locations in Malaysia, including remote islands (less than 200 km^2^) and decentralized places to identify possible wind energy applications. In this dissertation, extrapolation capabilities of the wind speed of the proposed hybrid ANFIS models have been examined for a location in Tioman Island having latitude 2° 48' 30" N and longitude 104° 8' 29" E, where measured wind data are not available. And then, the result is compared with the measured wind data at Mersing station (latitude 2° 27' N and longitude 103° 50' E) which is the nearby station having similar climate conditions. As the study location does not have any actual measured meteorological data, the daily average solar radiation (kWh/m2/day), daily average air temperature, maximum and minimum air temperature, air pressure, relative humidity and altitude data were collected from NASA surface meteorology and solar energy database for the whole year of 2004 for the prediction of daily average wind speed (m/s). The Figs 20 and 21 show the prediction of daily average wind speed for Tioman Island using ANFIS-PSO and ANFIS-GA respectively, which are compared with measured wind speed at Mersing meteorological station that was measured at 50m above the sea level in 2004.

From error analysis, it was found that the MAPE and MABE for ANFIS-PSO were 31.56%, 0.9672% respectively whereas, they were found to be 32.21% and 0.9872% for ANFIS-GA respectively. It can be observed from the figures that the wind characteristics in Tioman Island, obtained with the proposed ANFI-PSO and ANFIS-GA, is not exactly similar with the measured wind at Mersing in many cases. However, it is important to take note that the performance of the prediction models can be justified accurately when measured wind data in Tioman Island will be available for comparison.

## Conclusion

The wind energy potential assessment is very important for independent power producer and governmental organization to determine how efficiently wind power can be extracted from a certain location. The wind power density (WPD) is the key assessment parameter in wind potentiality analysis. Therefore, an efficient soft computing technique based on ANFIS-PSO, ANFIS-GA, ANFIS-DE and standalone ANFIS prediction models were developed in this paper to predict long-term (monthly and weekly) average wind power density of four different locations in Malaysia. The choice of the ANFIS technique was made due to its simplicity, reliability as well as its efficient computational capability; its ease of adaptability to optimization and other adaptive techniques, and its adaptability in handling complex parameters. The most significant advantage of hybrid ANFIS is that PSO/GA/DE tune the membership functions of the ANFIS model to ensure minimum error. The prediction models were trained and tested using wind speed data collected from meteorological stations of the underlying locations and measured wind power density. Moreover, different training and testing data size were applied to the prediction models to obtain best data size that provides a minimal error. The first 80% of data used for training and remaining 20% data for testing provide the optimal error in WPD prediction. Based on the result from best data size, there is no model that performed uniformly superior to other for all locations in both training and testing stages. Overall, ANFIS-PSO and ANFIS-GA out-performed ANFIS standalone and ANFIS-DE. Therefore, the results and analysis confirmed that the proposed hybrid ANFIS, especially ANFIS-PSO and ANFIS-GA have the excellent capability to predict the WPD with higher accuracy and precision. Other soft computing techniques applicable to wind speed and power density prediction for other parts of the world can be developed and compare with hybrid ANFIS in the further study.

## Supporting information

S1 Dataset Supporting Information.rar. (RAR)

## Abbreviations

ANFIS: Adaptive neuro-fuzzy inference system
ANN: Artificial neural network
DE: Differential evolution
ELM: Extreme learning machine
GA: Genetic algorithm
GP: Genetic programming
PSO: Particle swarm optimization
SVM: Support vector machine
GMCM: Gaussian mixture copula model
GPR: Gaussian process regression
WT: Wavelet transform
GS: Grid search
GHG: Greenhouse gases
MABE: Mean absolute bias error
MAPE: Mean absolute percentage error
RMSE: Root mean square error
R^2^: Coefficient of determination
MMD: Malaysian Meteorological Department
WPD: Wind power density
